# Ser/Thr protein phosphatases in fungi: structure, regulation and function

**DOI:** 10.15698/mic2019.05.677

**Published:** 2019-04-24

**Authors:** Joaquín Ariño, Diego Velázquez, Antonio Casamayor

**Affiliations:** 1 Departament de Bioquímica i Biologia Molecular and Institut de Biotecnologia i Biomedicina, Universitat Autònoma de Barcelona, Cerdanyola del Vallès, Barcelona, Spain.

**Keywords:** protein phosphorylation, protein phosphatases, cell signaling, S. cerevisiae, fungi

## Abstract

Reversible phospho-dephosphorylation of proteins is a major mechanism for the control of cellular functions. By large, Ser and Thr are the most frequently residues phosphorylated in eukar-yotes. Removal of phosphate from these amino acids is catalyzed by a large family of well-conserved enzymes, collectively called Ser/Thr protein phosphatases. The activity of these enzymes has an enormous impact on cellular functioning. In this work we pre-sent the members of this family in *S. cerevisiae* and other fungal species, and review the most recent findings concerning their regu-lation and the roles they play in the most diverse aspects of cell biology.

## INTRODUCTION

Phosphorylation of specific residues is a major mechanism for the control of the function of proteins in eukaryotes. Phosphorylation is catalyzed by protein kinases and these events are reverted by the action of protein phosphatases (PPases). As a common rule, the number of protein kinases exceeds in several fold the number of phosphatases. Thus, in the model yeast *Saccharomyces cerevisiae* there are only 43 protein phosphatases [1], in contrast to around 120 *bona fide* protein kinases. Quite often, the subcellular localization, substrate specificity, or activation state of PPases is determined by their interaction with other proteins, collectively called regulatory subunits. While it has been accepted in the past that activation of kinases is pivotal for regulation of signaling pathways, whereas PPases merely counteract the action of kinases, current evidence suggests that regulation of PPases could also play a key role in signal transmission [2, 3].

**Figure 1:**
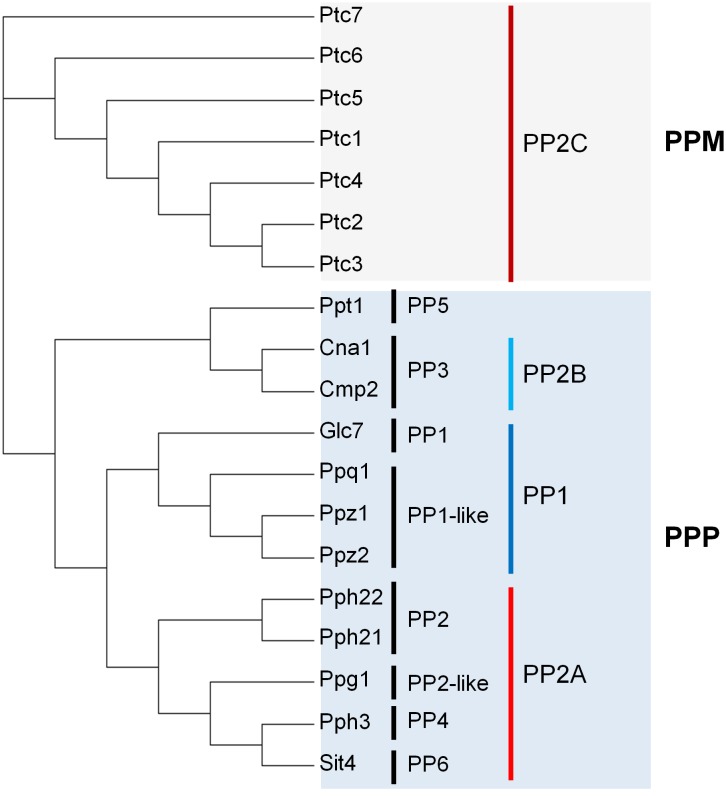
FIGURE 1: Phylogenetic analysis of protein phosphatase sequences from *S. cerevisiae* S*288c (taxid:559292).* The tree was constructed using the function "build" of Environment for Tree Exploration (ETE) v3.0.0b32 as implemented on the GenomeNet site (https://www.genome.jp/tools/ete/). The output files were imported to the open source Dendoroscope 3 software (v. 3.5.9). Protein sequences are described in Supplemental Table S1 (identified with the prefix “Sc_”).

In eukaryotes, most phosphorylation events occur at specific Ser and Thr residues and have both structural and signaling effects, while phosphorylation at Tyr, possibly accounting for only 1% of the phosphorylated sites, is mostly involved in signaling [4]. The genome of *S. cerevisiae* encodes 19 identified proteins with Ser/Thr protein phosphatase activity (**Figure 1**, see below). Twelve of these can be classified, based on their structure, into the PPP group. This includes the homologs of the widely distributed type 1 (PP1), 2A (PP2A) and 2B (PP2B) phosphatases that were identified in the early 1980s by biochemical approaches, mostly based on substrate specificity and sensitivity to thermostable inhibitors [5]. The remaining seven can be included into the PPM family and are likely homologs of the type-2C (PP2C) phosphatases, a kind of enzymes also characterized biochemically many years ago. It must be noted that PPP and PPM members are an example of convergent evolution, sharing a similar catalytic mechanism involving two metal ions at the catalytic site, but largely differing in primary sequence and tertiary structure.

If the PPP family in *S. cerevisiae* is considered, we can identify the expected equivalents of the type 1 (Glc7), 2A (Pph21 and Pph22), and 2B (Cna1 and Cna2) ubiquitous enzymes. These are, together with the type-2C proteins, what we define as "canonical" PPases. Besides, the *S. cerevisiae* PPP family includes additional members that are structurally closer to PP1 (Ppz1, Ppz2, Ppq1), PP2A (Pph3, Ppg1, Sit4), or PP2B (Ppt1). These proteins were identified by gene sequencing and are the ones we define here as "non-canonical". Experimental evidence for phosphatase activity has been obtained in most cases, at least for the *S. cerevisiae* enzymes, whereas in other yeasts or fungi it is often assumed on the basis of conserved structural features. In this work we will review both canonical and non-canonical Ser/Thr PPases in the yeast *S. cerevisiae*, with eventual references to the equivalent proteins from other fungi. This review largely focuses on the function and regulation of these enzymes, and therefore should effectively complement a very recent review by Offley and Schmidt [1], which is focused in *S. cerevisiae* only and emphasizes the structural and catalytic aspects. To note that, in contrast to the mentioned review, we do not include Ppn2 because, in spite of its somewhat distant relationship with PP2B protein phosphatases, this enzyme has been recently reported to be a Zn^2+^-dependent polyphosphatase [6] and, as far as we know, its protein phosphatase activity has not been proved.

## PP1 AND PP1-LIKE PHOSPHATASES

**Figure 2:**
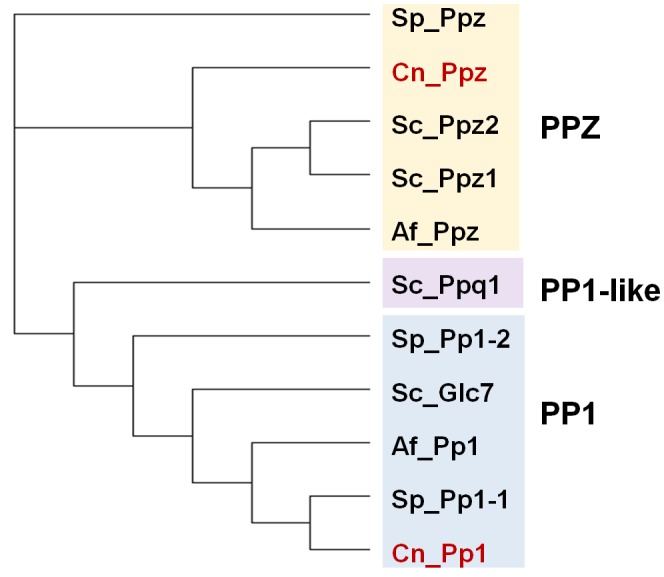
FIGURE 2: Phylogenetic tree of PP1 and PP1-like phosphatases from various fungal species. The protein sequences of the ascomycetes *S. cerevisiae* (Sc), *S. pombe* (Sp) and A*. fumigatus* (Af), as well as that of the basidiomycete *Cryptococcus neoformans* (Cn, in red) were used. Analysis was performed as in Figure 1. The corresponding sequence codes can be found in Supplemental Table 1.

In addition to the ubiquitous catalytic subunits of PP1 enzymes (PP1c), fungi contain two PP1-related PPases, Ppq1 and Ppz1, that are not found in other eukaryotes (**Figure 2**).

### PP1

Protein phosphatase-1 (PP1) was among the first biochemically characterized Ser/Thr phosphatases and it is probably the most extensively studied. Because the existence of relatively recent reviews [7, 8], we will provide here a general background and will then focus on the more recent findings.

In eukaryotes, PP1 is involved in many cellular functions including the regulation of glycogen metabolism, muscle physiology, RNA processing, protein synthesis, transmission of nerve signals, induction of apoptosis and control of multiple checkpoints, and events that occur throughout the cell cycle [8, 9]. To fulfill these roles, each functional PP1 enzyme consists of a catalytic subunit (PP1c) which binds to different proteins called regulatory subunits. These regulators are needed either to target the PP1 catalytic subunit to specific subcellular localization, to modulate substrate specificity or to serve as substrates themselves.

PP1c is highly conserved among all eukaryotes, with approximately 70% or greater sequence identity. Most fungal species contain one single gene coding for the PP1c, although in a few species, such as *Schizosaccharomyces pombe,* two genes are present. In the yeast *S. cerevisiae* this enzyme is encoded by a single gene, termed *GLC7* (aliases are *DIS2S1* and *CID1)*. For comparison, in mammals PP1c is encoded by three genes (PP1α, PP1β/δ and PP1γ), with two isoforms more (PP1γ1 and PP1γ2) which can be generated by alternative splicing. The name *GLC7* derives from the reduction in glycogen content identified in specific mutant strains [10–12]. As its mammalian counterparts, the functions of Glc7 are regulated by the interaction with different regulatory subunits affecting their substrate specificity and/or subcellular localization [8].

#### Structure

*GLC7* encodes an essential protein of 312 amino acids that is 85% identical to the four human PP1c proteins. The central section of Glc7 is also shared with the related yeast protein phosphatases PP2A, PP2B and Ppz1,2. Orthology of human PP1 isoenzymes with Glc7 has been verified by complementation of a *glc7* mutant with human PP1c cDNAs [13].

There are currently more than twenty 3D-structures available of the mammalian PP1 catalytic subunit. PP1c adopts a compact α/β fold, with a β sandwich wedged between two α-helical domains, which are the C-terminus, and the extreme N-terminus of the protein. The β sandwich and the two helical domains form a “Y”-shaped cleft where the active site is located. There, an invariant number of residues (three His, two Asp and one Asn) coordinate two metal ions, Mn^2+^ and Fe^2+^, which are needed to contribute to catalysis. These residues are highly conserved in all members of the PPP family suggesting a common mechanism of metal-catalyzed reaction [14]. Through that cleft, there are three grooves called hydrophobic, acidic and C-terminal.

#### Regulation and binding motifs

PP1c is a relatively small protein, which does not exist freely in the cell. It achieves its huge functional diversity by interacting with a large variety of structurally unrelated regulatory subunits, with distinct effects on the function of the phosphatase. More than 100 putative PP1 regulatory subunits have been described in mammals, whereas the yeast Glc7 phosphatase associates with around 30 of these proteins [1, 8].

Despite their apparent differences in sequence, most of these subunits bind to PP1c in the same manner. Binding to PP1c is mediated by docking motifs, that are short sequences of about 4-8 residues present in the regulatory subunits that are combined to create a larger interaction surface for PP1c. Despite the conservation of motifs during evolution, they are somewhat degenerated, displaying variants of the consensus sequence that differ in affinity for PP1c. There are about ten known distinct PP1-docking motifs identified in the regulatory subunits in mammals, although not all of them are found in yeast. Most regulatory subunits bind to PP1c by the identifiable RVxF consensus sequence using the hydrophobic groove as PP1c interface (see [9] for a review). Mutation of residues of this hydrophobic groove reduced affinity to some regulatory subunits, resulting in phenotypic traits related to reduced Glc7 activity [15], and several of these variants could not restore viability in a *glc7* deletion mutant [8]. Among the regulatory subunits for which the RVxF motif is key for interaction are Ref2, Gip2, Afr1, Reg1, Reg2, Sla1, Bud14, Bni4 and Gac1. In some cases, more than a putative consensus is found (i. e. in Scd5, Gip1 or Fin1), although not necessarily all of them are required for interaction [16]. In contrast, Sds22 interacts with Glc7 at a region different from the hydrophobic groove and using a different motif that is based, similarly to its mammalian counterpart, in the characteristic leucine-rich repeats [17, 18]. In the case of Pti1, an essential component of the CPF (cleavage and polyadenylation factor) that interacts with Glc7, the interaction motifs are unknown, but likely are not based on the RxVF consensus [19]. Finally, certain regulatory subunits can associate to form larger complexes. For instance, formation of a trimeric complex involving Glc7 together with Sds22 and Ypi1 has been reported as necessary for translocation of the phosphatase to the nucleus [20]. In this complex both regulatory subunits interact with Glc7 at different sites and the formation of the trimeric complex reinforces bipartite interactions.

#### Functions

**Figure 3:**
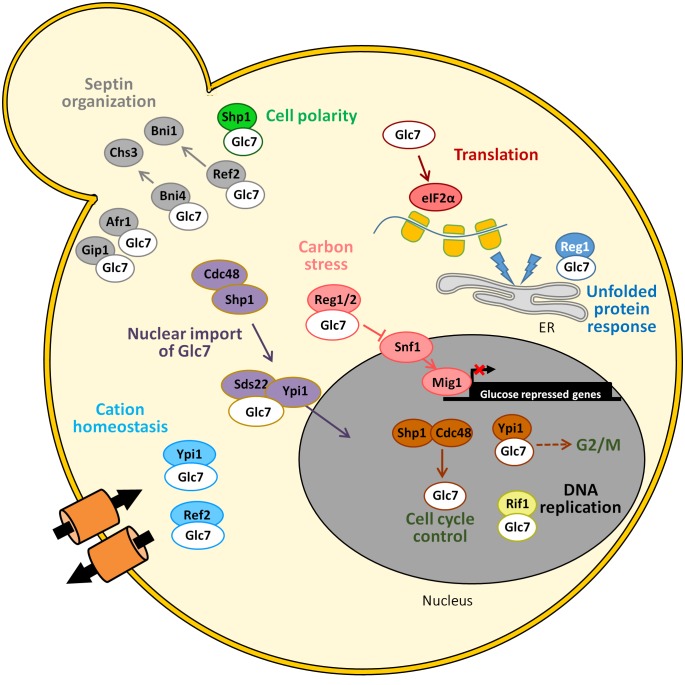
FIGURE 3: Schematic depiction of the diverse associations of Glc7 with its regulatory subunits and the functions in which the complexes are involved. See main text for details.

Glc7 can play very diverse roles role in different cellular localizations in yeast cells (**Figure 3**). Cytosolic functions for Glc7 related to glucose repression, regulation of septins assembly and chitin synthesis, bud site selection or endocytosis and actin organization, as well as nuclear tasks, related to transcriptional regulation (dependent and independent of the CPF complex), microtubule attachment to kinetochores, and diverse cell cycle checkpoints have been nicely reviewed in detail several years ago [8]. Therefore, we will only summarize here some of the more recent findings.

In addition to Reg1, its paralog Reg2 has been found to assist Glc7 in the dephosphorylation of the protein kinase Snf1 in the presence of high glucose concentrations [21, 22]. Although Reg1-Glc7 regulates dephosphorylation of all three Snf1 isoforms, it preferentially associates with the one containing the Gal83 subunit [23], the only one capable of nuclear localization upon glucose limitation. Moreover, the tandem Glc7-Reg1 may also be important for inactivation of genes dispensable for growth in high glucose, such as *HXK2, PDA1* and *HSP60* [24]. Although Reg1-Glc7 plays the major role in the Snf1-mediated signaling pathway, other phosphatases like Ptc1 or Sit4 contribute to maintenance of the Snf1 activation loop in the dephosphorylated state during growth on high glucose [25, 26]. A major target for Snf1 is the Mig1 repressor, whose phosphorylation promotes its eviction from the nucleus. A possible role for the Glc7-Reg1 phosphatase in the direct dephosphorylation of Mig1 was also hinted several years ago [27]. More recently, the possibility of the existence of an additional glucose and Glc7-Reg1 independent mechanisms for dephosphorylating Mig1, perhaps involving Tyr phospho-dephosphorylation, has been suggested [28].

Glc7 also has relevance in the unfolded protein response (UPR). It has been reported that lack of Reg1 causes hypersensitivity to UPR inducers, which is concomitant with an augmented UPR element-dependent transcriptional response. These effects are attributable to the inappropriate activation of Snf1 [29].

As mentioned above, Glc7 is important in several cell cycle check-points. The lack of the essential regulatory subunit Ypi1, the yeast homologue of mammalian inhibitor 3, activates the morphogenetic checkpoint. Depletion of Ypi1 results in stabilization of the Pds1 securin, suggesting the activation of a G2/M checkpoint [30]. Since under normal conditions most of the Glc7 protein is found in the nucleus, in particular in the nucleolus, it is possible that these defects could be due to the alteration of its nuclear localization previously reported for Ypi1-depleted cells [20]. Shp1, a protein involved in shmoo formation and bipolar bud site selection, could also be a positive regulator of Glc7, working in a complex with the AAA-ATPase Cdc48 and promoting cell cycle progression [31]. Nuclear localization of Glc7 requires the Cdc48-Sph1 complex, which possibly functions as a molecular chaperone for the structural integrity of the PP1 complex, and specifically promotes the assembly of Glc7-Sds22-Ypi1 for nuclear import [32] (**Figure 3**).

The subunits Bni4, Afr1 and Gip1 mediate different septin localization activities. Specifically, the Glc7-Bni4 holoenzyme regulates the targeting of chitin synthase III (Chs3) to the incipient bud sites when Bni4 is phosphorylated by the Pho85 kinase-Pcl1,2 cyclin complex [33]. It is worth noting that depletion of Ypi1 also causes depletion of the Cdc11 septin, which possibly explains the failure to form properly assembled septin rings at the bud necks [30].

Early work linked Glc7 to maintenance of monovalent cation homeostasis [34]. More recently, two Glc7 subunits have been found relevant for tolerance to toxic cations. Thus, lack of Ref2 is additive to blockage of the calcineurin pathway and might disrupt multiple mechanisms controlling expression of the *ENA1* Na^+^-ATPase-encoding gene in a way dependent on Glc7 but independent of its previously known function in the formation of mRNA via the APT (for Associated with Pta1) subcomplex of the large CPF complex [35]. Remarkably, partial depletion of the aforementioned Ypi1 subunit renders cells sensitive to Li^+^ and this phenotype is due, at least in part, to an increased expression of *ENA1*. It appears, however, to be independent of the role of Ypi1 as a Glc7 regulatory subunit and, instead, the increase Li^+^ tolerance and *ENA1* expression is abolished in a *cnb1* mutant, indicating that the effect of Ypi1 on cation homeostasis is essentially mediated by calcineurin [36]. More recently, it has been postulated that Ref2-Glc7 would be required for dephosphorylation of the formin Bni1, thus playing a role in defining the subcellular localization of formins during cytokinesis [37].

Recently, Rif1 was identified as a new possible regulatory subunit of Glc7. The Rif1 protein, originally identified as a telomere-binding factor in yeast, was also found to prevent premature activation of replication origins in late-replicating chromosomal domains. This is achieved by targeting Glc7 to the sites of action to direct the dephosphorylation of subunits of the Minichromosome Maintenance (MCM) complex [38] and could be important in the replication of the highly repetitive and highly transcribed rDNA locus [39]. It has been suggested that Rif1 interacts with Glc7 through two ‘SILK' and two ‘RVxF' putative consensus sequences located at its N-terminus [38]. Regulation of replication by Rif1 through PP1c is an evolutionarily conserved mechanism. Recent work has provided evidence that control of telomere length by Rif1 also involves targeting of Glc7, and that this role is independent of its effect on replication timing (see [40] for a recent review).

The eukaryotic translation initiation factor 2 (eIF2), is required for initiation of translation. eIF2 is composed of α, β, and γ subunits and translation initiation requires dephosphorylation of the α subunit at Ser51. Although Glc7 was identified long ago as a major eIF2α phosphatase, in *S. cerevisiae* it was not evident which regulatory subunits are relevant for targeting the phosphatase to eIF2α, as these were not known in mammalian cells. Only recently, Rojas and coworkers [41] nicely demonstrated that, in fact, such subunit might not exist, and that it would be replaced by the eIF2γ component of the complex, by means of a RVxF-like motif (KKVAF) present in its N-terminal extension. Such motif would be rather unique to certain yeast species, which would rely on the recruitment of PP1 in *cis* to the eIF2 complex to maintain eIF2α phosphorylation at the appropriate levels.

#### Role of Glc7 in virulence in Candida albicans

Virulence in *Candida albicans,* one of the most common fungal pathogen in humans, is largely linked to its ability to switch from yeast to hyphal forms [42]. Interestingly, some PP1c regulatory subunits or substrates have been related to virulence in this organism. For instance, it has been found that Sds22 plays an important role in Rad53 dephosphorylation and, therefore, in deactivation of the DNA damage checkpoint, through inhibitory physical association with Glc7 [43]. These same authors showed that overexpression of *SDS22* reduces *C. albicans* virulence in a mouse model of systemic infection.

Deletion mutants for *Cas5*, encoding a transcriptional regulator of genes involved in cell wall integrity that has no orthologue in *S. cerevisiae*, display attenuated virulence and enhanced sensitivity to the antifungal fluconazole. Recent work has shown that Cas5 is activated by Glc7 in response to cell wall stress, playing a role not only in cell wall homeostasis but also in regulating nuclear division [44].

### PPQ1

The gene *PPQ1* encodes a type1-related phosphatase of 549 residues in length. The C-terminal half contains the phosphatase domain, whereas its N-terminal extension is rich in Ser and Asn residues (although unrelated in sequence to Ppz1/2 phosphatases, see below). The protein is not conserved in other eukaryotes and it is t even absent in many fungal species. The gene was initially isolated (and named *SAL6*) as an allosuppressor able to enhance the efficiency of omnipotent suppressors thought to be translational ambiguity mutations [45], and a few years later cloned by sequence homology and by complementation of the *sal6-1* mutation. These initial studies (see [46] and references therein) already prompted about a possible role of Ppq1/Sal6 in protein translation, still unknown, although subsequent studies showed that Sal6 does not dephosphorylate the eukaryotic release factor eRF1 [47].

Little advance was made for quite a few years on the functional role of Ppq1. Only recently, metabolomic studies using kinase and phosphatase mutants attributed a role of Ppq1 in metal homeostasis (mainly Mn^+2^) which would affect the activity of the tricarboxylic acid (TCA) cycle [48], although this issue has not been investigated further. Interestingly, Ppq1 was also identified as a phosphatase able to down-regulate the mating signaling pathway by targeting at or upstream of the terminal MAP kinase Fus3 [49]. Such role was confirmed by an independent laboratory using a phosphatase overexpression strategy, placing the MAPKK Ste7 and the MAPK Fus3 as possible targets [50]. During the study of the role of the Cdc48–Shp1 complex in regulating nuclear targeting of Glc7 and promotion of the assembly of the Glc7–Sds22–Ypi1 PP1 complex, it was found that Ppq1 also associates with Shp1 and forms aggregates in Shp1-depleted cells upon proteasome inhibition [32]. Ppq1 has been found to interact with Ypi1 and Sds22 as well [51] and, similarly to Glc7, the interaction with Sds22 is necessary to permit the association between Shp1 and Ppq1 [32]. This suggests that Cdc48–Shp1 might have a general role in the assembly of PP1-like phosphatases containing Sds22 and Ypi1.

### PPZ Phosphatases

#### Structure

The Ppz phosphatases are enzymes apparently restricted to fungal species and characterized by a well-conserved carboxy-terminal domain, related to type 1 PPases, and a N-terminal domain that largely differs in sequence and size among fungi. These enzymes were first identified in *S. cerevisiae*, where two paralogs, *PPZ1* and *PPZ2* are found [52, 53]. In this yeast Ppz enzymes display a C-terminal catalytic domain of about 300 residues, which is 75-90% identical to other fungal Ppz enzymes and retains ~60% identity with PP1 catalytic subunits. The N-terminal moieties of Ppz1 and Ppz2 are roughly of the same size (~ 350 residues), but they are more divergent in sequence. Still, they include a conserved Gly2 that is myristoylated *in vivo* [54], possibly due to the action of the Nmt1 N-myristoyl transferase. In addition, a relatively conserved sequence near the N-terminus of Ppz1 and Ppz2 (^43^SSRSRRSLPS^52^ and ^43^SSRSLRSLRS^52^, respectively) can be found in many fungi in the form of a SxRSxRxxS consensus [55]. Such sequence seems to have functional relevance (see below). Besides this, the N-terminal half of Ppz proteins exhibits low or very low conservation among fungi and is often shorter than that found in *S. cerevisiae*. Ppz1 is recovered in particulate fractions from yeast extracts [54] and different studies have shown that, at least in part, is localized at the cell periphery [32, 56].

#### Function

Deletion of *S. cerevisiae PPZ1* results in many phenotypic traits, whereas that of *PPZ2* is hardly noticeable, suggesting that the former enzyme has a more prominent cellular role. However, deletion of *PPZ2* in a *ppz1* background usually potentiates the phenotypes. Ppz1 has a major role in salt tolerance, and strains lacking Ppz1 are hypertolerant to sodium or lithium cations, a phenotype enhanced by additional deletion of *PPZ2* [57]. Such tolerance, at least in part, is the result of an increase in the expression of the *ENA1* ATPase gene, whose levels are induced by salt stress and alkaline pH and represents a major determinant for sodium tolerance in budding yeast. Thus, the effect of Ppz1 on *ENA1* expression is opposite (see below) to the effect described for the Ser/Thr phosphatase calcineurin, a positive effector of the *ENA1* gene [58]. In fact, it has been shown that the effect of the absence of Ppz1 on *ENA1* expression requires an intact calcineurin pathway [59], thus suggesting that Ppz1 negatively regulates calcineurin activity.

Nevertheless, Ppz1 also influences salt tolerance in an *ENA1*-independent way. Early evidence came from the observation that overexpression of the Sky1 protein kinase increases sensitivity to LiCl in a way that requires the function of *PPZ1* but not that of *ENA1* [60]. Shortly afterwards, it was demonstrated that Ppz1 was a negative regulator of potassium influx through the high-affinity potassium transport system encoded by Trk1 and Trk2 [61]. Indeed, cells lacking *PPZ1* and *PPZ2* showed increased potassium uptake, leading to augmented intracellular turgor. This effect could explain the impact of Ppz1 on the cell wall integrity (CWI) pathway and provided the basis to understand earlier findings pointing to the involvement of Ppz1 and Ppz2 in the maintenance of CWI, such as the fragility of *ppz1 ppz2* mutants in the presence of caffeine, unless osmotically stabilized [62], and the isolation of *PPZ2* as a high-copy suppressor of the lytic phenotype of *slt2*/*mpk1* and *pkc1* mutants, lacking key components of the CWI pathway [53]. It is not known how Ppz phosphatases influence Trk-mediated K^+^ transport, but it has been shown that Trk1 physically interacts with Ppz1, and that the *in vivo* phosphorylation level of Trk1 increases in a Ppz-deficient strain [56]. However, no experimental evidence for direct dephosphorylation of Trk1 by Ppz1 has been obtained. The Ppz phosphatases also regulate potassium influx in a Trk-independent way, which involves calcium signaling but not calcineurin activation [63]. Interestingly, Ppz1 down-regulated the contribution to K^+^ influx of an heterogously expressed barley HvHak1 transporter (a kind of K^+^ transporter also present in some fungi but not in *S. cerevisiae* [64]), thus raising the possibility that the regulatory network controlling K^+^ homeostasis in fungi could be conserved. The impact of Ppz-phosphatases on cation homeostasis likely lays on the basis of a number of reported phenotypes: enhanced tolerance to toxic cations, such as Hygromycin B, tetramethylammonium or spermine [61, 63, 65], sensitivity to agents causing replicative stress or DNA damage [66], formic acid susceptibility [67] or even modulation of flocculation and invasive growth phenotypes [68].

Recent evidences have linked Ppz phosphatases to the regulation of ubiquitin homeostasis, possibly by controlling the phosphorylation state of ubiquitin at Ser57, and it was porposd that the salt–related phenotypes of the *ppz* mutants are related to ubiquitin deficiency [69]. Even more recently, the ubiquitin ligase adaptor Art1 has been recognized as a Ppz substrate. In this role, that would be distinct from that played on ubiquitin, Ppz would mediate the methionine-induced dephosphorylation of Art1. Such dephosphorylation would promote cargo recognition, in this case that of the methionine transporter Mup1, at the plasma membrane [70].

The Ppz phosphatases are also likely influencing protein translation. Thus, it was demonstrated that Ppz1 interacts *in vivo* with translation elongation factor 1Bα (Tef5), the GTP/GDP exchanging factor for translation elongation factor 1, and that in *ppz1 ppz2* cells the conserved Ser86 of Tef5 was hyperphosphorylated. Indeed, lack of Ppz phosphatases resulted in enhanced read-through at all three nonsense codons, suggesting that translational fidelity might be affected [71]. A role of Ppz1 (and possibly Ppz2) on protein translation accuracy has been reinforced by evidences of its role in the regulation of read-through efficiency and manifestation of non-Mendelian anti-suppressor determinant [ISP(+)] [72].

#### Regulation

*In S. cerevisiae*, Ppz1 is regulated *in vivo* by Hal3 (Sis2), encoded by a gene originally identified as a high-copy suppressor of the cell cycle-related growth defect of a strain lacking the Sit4 phosphatase [73] (also reviewed in this work), and by its capacity to confer halotolerance [74]. Hal3 binds to the carboxyl-terminal catalytic domain of Ppz1 and strongly inhibits its phosphatase activity, thus modulating its diverse physiological functions [75]. For instance, cells overexpressing Hal3 are salt-tolerant, whereas a *hal3* strain is hypersensitive to sodium and lithium cations. Likewise, high-copy expression of *HAL3* exacerbates the lytic phenotype of a Slt2 MAP kinase mutant whereas, in contrast, lack of *HAL3* improves growth of this strain [75]. The effect of Hal3 overexpression on cell cycle was also shown to depend on Ppz1 function, as deduced from the observation that mutation of *PPZ1* rescues the synthetic lethal phenotype of *sit4 cln3* mutants [76].

This general effect of the regulatory subunit Hal3 on Ppz1 function appears rather different from the situation described for Glc7. Deletion of *GLC7* results in lethality [10, 11] whereas the absence of regulatory components yields less dramatic phenotypes (only three of them, Scd5, Sds22 and Ypi1 are also essential in *S. cerevisiae*), suggesting that the diverse cellular roles attributed to Glc7 are the result of specific interactions of the catalytic subunit with different regulatory subunits [8]. It must be noted, however, that Ppz1 and Glc7 might not be fully insulated with respect to some specific functions or to modulation by their counterpart regulators. For instance, *PPZ1* and *PPZ2* display genetic interactions with *GLC7*, as deduced from the different growth defects observed in cells carrying certain mutant alleles of *GLC7* in combination with null alleles of the PPZ phosphatases [77]. As mentioned above, many (about 2/3) of PP1c (and Glc7) regulatory subunits contain a RVxF consensus PP1c binding motif [78], which binds to a hydrophobic groove strongly conserved in Ppz1. It is worth noting that *in vivo* interactions between Ppz1 and two Glc7 regulatory subunits displaying RVxF motifs (Glc8 and Ypi1), has been reported by 2-hybrid analysis [77]. Interaction between Ppz1 and Ypi1 has been also documented by pull-down assays (although Ypi1 barely affects Ppz1 activity), and it was shown that a W53A mutation in its RVxF motif (^48^RHNVRW^53^) abolished binding to both the Glc7 and Ppz1 phosphatases [79]. In addition, both *S. cerevisiae* and *C. albicans* Ppz1 are sensitive *in vitro* to mammalian Inhibitor-2 [80, 81], a PP1c regulatory subunit that contains a ^144^RKLHY^148^ sequence functionally replacing the RVxF motif. These observations suggested that the RVxF-binding motif is also functionally conserved in Ppz1.

The Ppz1 inhibitor Hal3 contains a ^263^KLHVLF^268^ sequence alike to the RVxF motif. However, mutation of H^265^ or F^268^ does not affect binding nor inhibitory capacity of Hal3 upon Ppz1 [82], suggesting that this RVxF-like motif is not relevant for the interaction with Ppz1. Sequence comparisons and recent experimental evidence on the *C. albicans* Ppz1 C-terminal domain [81] indicate that diverse docking motifs found in PP1c, such as PNUTS or spinophilin, are likely not relevant for yeast Ppz1. The structural determinants for interaction between Ppz1 and Hal3 are still unknown, but they should differ substantially from those used by PP1c-regulatory subunits to bind to PP1c, since Hal3 does not bind to Glc7 *in vitr*o [75, 79]. In any case, Ppz1 and Hal3 can be co-expressed in *Escherichia coli* and purified as a complex with an apparent 1:1 stoichiometry [83], and a recent study has suggested that inhibition of Ppz1 by Hal3 could happen by occlusion of the catalytic site, in a way similar to that used by inhibitor-2 to inhibit PP1c [84]. In *S. cerevisiae* it has been postulated that the interaction between Ppz1 and Hal3 is dependent on the internal pH and serves to maintain intracellular pH homeostasis [56].

The *S. cerevisiae* genome contains a paralog of Hal3, named Vhs3, which was identified as a high-copy suppressor of the synthetically lethal phenotype of the *hal3 sit4* mutation [85]. Vhs3 also inhibits Ppz1 *in vitro*, although its role regulating the phosphatase *in vivo* is far less important, probably due to lower expression levels [86]. Remarkably, in *S. cerevisiae* the simultaneous deletion of *HAL3* and *VHS3* is synthetically lethal, and this is not due to hyperactivation of Ppz1 [86]. Such interaction was explained by the discovery that Hal3 and Vhs3 are moonlighting proteins. Thus, Hal3 and/or Vhs3 associate with Cab3 (also a Hal3 and Vhs3 paralog) to form an active, heterotrimeric phosphopantothenoylcysteine decarboxylase (PPCDC) enzyme [87]. PPCDC is an essential enzyme that catalyzes a key decarboxylation step in Coenzyme A (CoA) biosynthesis. While in most organisms PPCDC is an homotrimer with three catalytic sites, each formed at the interface of two monomers, in budding yeast a single catalytic site is formed at the interface of Cab3 and either Hal3 or Vhs3, thus explaining the essential nature of *CAB3* and the synthetically lethal phenotype of the *hal3 vhs3* mutations [87]. It has been proposed that Vhs3 has a higher tendency to form heterotrimers, whereas Hal3 can be easily released and undergo monomer exchange, thus becoming able to interact with Ppz1 [83].

The subunit composition of *S. cerevisiae* PPCDC is rather exceptional, not only because in most eukaryotic organisms, such as humans and plants, PPCDC is an homotrimer, but also because this unique component subunit is a much shorter polypeptide (~250 residues), lacking the N-terminal extension and the large acidic C-terminal tail also found in certain fungal orthologs, such as *C. albicans* [88]. Previous studies have shown that this central domain, denoted as Hal3 PD, is required for Ppz1 binding and regulation, although the acidic C-terminal tail also plays an important functional role [89]. Full-length Hal3 (as well as the PD domain) can form trimers itself. This ability is altered by mutation of L405 to Glu, which would disrupt a possible hydrophobic core in the trimer, although the change does not abolish the ability to interact with Cab3 and to generate a functional PPCDC *in vivo*. Remarkably, this mutation decreases binding with Ppz1 *in vitro* and causes partial loss of Ppz1-mediated functions *in vivo* [90].

#### Ppz1 phosphatases in other fungi: relevance for virulence

Ppz1 has been also characterized in diverse fungi, where usually only a single gene is found. The PZL-1 phosphatase from the filamentous fungus *Neurospora crassa* was able to replace *S. cerevisiae* Ppz1 in diverse phenotypic tests related to cation homeostasis and interaction with the CWI pathway [91]. *C. albicans* Ppz1 (CaPpz1) also behaves similarly to ScPpz1 albeit with some characteristic traits [92], whereas in the fission yeast *S. pombe* the single Pzh1 was shown to regulate cation homeostasis, but with distinct characteristics in comparison with budding yeast [93, 94]. In the halotolerant yeast *Debaryomyces hansenii*, DhPPZ1-deficient strains were salt tolerant, but the effect was found related to the Na^+^/H^+^ antiporter [55].

In the last few years, the focus has been placed on the enzyme from pathogenic fungi. The *Aspergillus fumigatus* ortholog *phzA*, when overexpressed in *S. cerevisiae*, mimicked in part the role of ScPpz1. In contrast, the *A. fumigatus* mutant did not display altered salt tolerance or CWI defects, but exhibited sensitivity to oxidant agents [95]. Further work confirmed the sensitivity to oxidative stress and found PhzA to be relevant for iron assimilation, conidiation and virulence [96, 97]. More recently, it has been reported that this mutant (named here *ppzA)* has decreased production of diverse siderophores and other secondary metabolites, which might be linked to the fact that these mutants are avirulent in a murine infection model [98].

The enzyme from *C. albicans* was cloned, functionally characterized, and found to be relevant for virulence [92, 95, 99]. The catalytic domain of CaPpz1 has been crystallized and its 3D-structure solved [81], providing insights into unique Ppz1 features that could be useful for antifungal drug design. Recent evidence suggests that, as it was demonstrated for *ScPpz1*, the N-terminal domain of CaPpz1, although much shorter, is functionally relevant [100]. *C. albicans* contain two genes, orf19.3260 and orf19.7378, encoding putative homologs of ScCab3 and ScHal3, respectively [88]. Remarkably, whereas both CaHal3 and CaCab3 retain their predicted PPCDC-related functions (thus likely generating a heterotrimeric PPCDC), only CaCab3 was able to regulate CaPpz1 *in vivo.* Therefore*,* CaCab3, but not CaHal3, acts as a moonlighting protein in *C. albicans* [88]. A recent proteomic analysis provided further support to the idea that Ppz phosphatases might be related to protein translation in fungi [101].

Very recent work has characterized the functions of Ppz1 in the pathogenic fungus *Cryptococcus neoformans* and found that the phosphatase could only partially complement a *S. cerevisiae ppz1* deletion mutant and was not involved in virulence using a *Galleria mellonela* infection system [102]. Remarkably, *C. neoformans* encodes two similar Hal3-like proteins, CnHal3a and CnHal3b. Both of them act as PPCDC, but none is able to regulate Ppz1 functions *in vivo* nor inhibit the phosphatase *in vitro* [102], indicating that the inhibitory properties of Hal3-like proteins are not conserved across the fungal kingdom. Therefore, Hal3 proteins do not perform moonlighting tasks in *C. neoformans.* Deletion of the gene encoding CnHal3b renders cells less virulent [102]. No impact on virulence has been determined for the plant fungal pathogen *Fusarium graminearum*, the causative agent for wheat scab [103]. Therefore, involvement of Ppz1 in virulence seems not to be a general issue, but rather species-specific.

## PP2A AND PP2A-LIKE PHOSPHATASES

**Figure 4:**
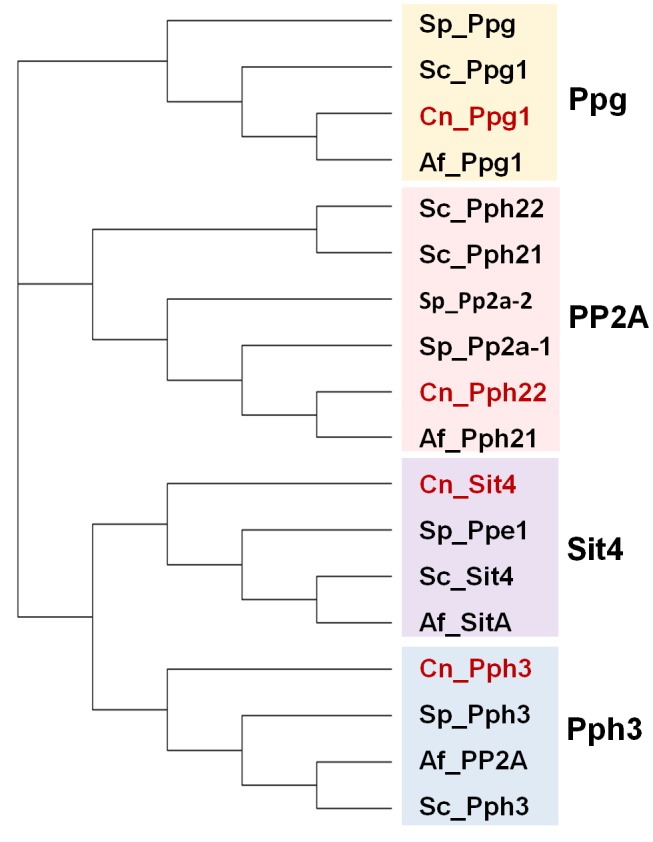
FIGURE 4: Phylogenetic tree of PP2A and PP2A-like phosphatases from various fungal species. Protein sequences correspond to organisms described in the Figure 2. The analysis was performed as described in Figure 1.

The family of the catalytic subunits of PPases type 2A and 2A-like in fungi comprises the canonical PP2A and the non-canonical Sit4, Pph3 and Ppg proteins (**Figure 4**).

### The PP2A phosphatases

The PP2A phosphatases are present in all organisms and their structure is conserved across eukaryotes. They are involved in many and essential processes, including cell growth, differentiation, apoptosis, cell motility, DNA damage response and cell cycle progression [104].

#### Structure

PP2A can be found as a heterotrimeric complex, composed of a C catalytic subunit (PP2Ac), a scaffolding A subunit, and a B regulatory subunit which is thought to determine the substrate specificity, as extensively reviewed [105]. Although only one gene coding for PP2Ac is present in most fungi, this enzyme is encoded by two genes in *S. cerevisiae*: *PPH21* and *PPH22*. These catalytic polypeptides are highly conserved, as exemplified by the fact that the catalytic cores of the *S. cerevisiae* Pph21 (from amino-acid 9 to the C-terminal) and human PP2Aβ (AAV38333.1, from amino-acid 69 to the C-terminal) share 75.4% of their residues, and 87.7% are similar. Deletion of both *S. cerevisiae* genes affect vegetative growth, and cells cannot survive if the yeast PP4 gene (*PPH3*) is also deleted [106]. Thus, Pph3 can perform, at least, the essential functions of Pph21/Pph22. The A subunit, encoded by the *TPD3* gene in *S. cerevisiae* and by *PAA1* in *S. pombe,* contains multiple HEAT repeats and is required for association to the catalytic C subunit. Although mammals express a combination of several splicing alternatives of diverse variable B regulatory subunits (classified in four main gene families), the alternative regulatory subunits in yeasts are reduced to a 55 kDa regulatory B subunit (Cdc55 in *S. cerevisiae*, Pab1 in *S. pombe*), the 56 kDa B' subunit (Rts1 in *S. cerevisiae*, Par1 and Par2 in *S. pombe*), and a *Saccharomycetales*-specific predicted B-subunit (Rts3 in *S. cerevisia*e). Pph21/Pph22 regulatory proteins can also bind to non-canonical/atypical PP2Ac-like proteins, such as Sit4 (reviewed in [107]).

#### Regulation

Several residues in Pph21 and Pph22 can be covalently modified by reversible phosphorylation and methylation, thus regulating the ability to form PP2A heterotrimers [108]. As other PP2A, it has been detected that yeast Pph21 is phosphorylated in the Tyr367 residue of the conserved C-terminal sequence TPDYFL [108]. Mutagenesis studies determined that phosphorylation of either this Tyr or Thr364 within the conserved motive, decreases the binding to Cdc55 [109]. Ppm1 was identified as the methyltransferase that catalyzes the methylation in *S. cerevisiae* of the carboxyl terminal Leu369 of PP2A, whereas Ppe1 catalyzes its demethylation [110]. Tap42 (**T**wo A Phosphatase **A**ssociated **P**rotein) together with Tip41 (**T**ap42 **I**nteracting **P**rotein of 41 kDa) acts as an inhibitor of the PP2A proteins and, in the presence of a good nitrogen source, TOR proteins promote the formation of the Tap42-PP2Ac complex. Binding of PP2Ac to Tap42 and Tpd3 is mutually exclusive [111]. The yeast PP6 protein Sit4 can also be found as a complex with Tap42 (see below). Rrd1 and Rrd2, also known as Ypa1 and Ypa2, are PP2A and PP2A-like positive regulators, and belong to the widely distributed phosphotyrosyl phosphatase activator (PTPA) family of proteins. Rrd1,2 are involved in the regulation of the TOR pathway [112, 113].

Phosphorylation of PP2A regulatory subunits is another mechanism for regulation of PP2A activity. Several examples are known in mammals, with different effects on PP2A activity (recently reviewed in [114]). Multiple phospho-Ser/Thr residues have been identified in yeast Cdc55 and Rts1. For example, Rts1 is phosphorylated in its Thr242 by the Cdk Cdc28 [115].

PP2A^Cdc55^ can be inhibited by the conserved Igo/ENSA endosulfine domain-containing proteins, frequently localized in the nucleus. In *S. cerevisiae* this family is represented by the pair of paralogous Igo1 and Igo2, although in other fungi only one protein exists (see also below). The *Saccharomycetales*-specific Zds1 and Zds2, a pair of redundant paralogs, localized in the cytoplasm and on the sites of cell polarity, are also negative modulators of PP2A^Cdc55^ that can be considered as regulators of the PP2A^Cdc55^ complex localization. Zds2 protein directly binds to the Cdc55, Tpd3 and Pph21 subunits of PP2A but its direct binding to Pph22, identified as part of the same complex, has not been detected [116]. No direct or indirect interactions have been identified between Zds proteins and the 56 kDa B' regulatory subunits (Rts1 or Par1/Par2) neither in *S. cerevisiae* nor in *S. pombe*, according to the Biogrid database (v. 3.5). The difference in localization suggests that Igo1/2 and Zds1/2 proteins control distinct functions of PP2A^Cdc55^ and do so by different mechanisms. Zds proteins, however, play a major role in the inhibition of PP2A^Cdc55^ in early mitosis, when compared to the endosulfine proteins [117].

The recently characterized STRIPAK (STRiatin-Interacting Phosphatases And Kinases), an eukaryotic protein complex highly conserved in animal and fungal species, could also be considered as a regulatory mechanism for PP2A proteins [118]. First identified in human, striatin orthologs have been found in all fungi: Far8 in *S. cerevisiae*, Csc3 in *S. pombe* or HAM-3 in *N. crassa*. The STRIPAK-like complexes in *S. cerevisiae* (also called yeast FAR complex) comprises, in addition to the Far8 striatin protein, PP2Ac and its scaffolding regulatory subunit, Tdp3, together with Far3, Far7, Far10, Far11 and Vps64/Far9. No direct physical interaction has been detected between *S. cerevisiae* Far8 and any PP2Ac, according to the BioGrid database, but direct physical interactions of *S. cerevisiae* Far11 with Pph21, Pph22, Pph3 and Tpd3 have been identified [119]. In *S. pombe*, Csc3 does not interact either to Ppa1 or Ppa2, but it does with the PP2A-related Ppa3 (Ppg1 in *S. cerevisiae*). A major biological role for the Far complex in *S. cerevisiae* is the pheromone-induced cell cycle arrest, although other functions, such as regulation of spatial cell growth by antagonizing TORC2, have been reported [119]. The STRIPAK-like complex in *S. pombe* has been implicated in the regulation of septation, being an inhibitor of the Septation Initiation Network (SIN) [120].

PP2A could also be regulated by the type 1 protein phosphatase, as was described in the fission yeast, whereby PP1 binds to and activates PP2A^Pab1^ through a conserved RVXF motif present in the B55 subunits. Active PP2A^Pab1^ dephosphorylates Par1 and promotes PP1 recruitment to activate the PP2A^Par1^ phosphatase. In this model, that could be valid for other organisms, PP1-induced activation of both PP2A^B55^ and PP2A^B56^ coordinates mitotic progression and exit [121].

#### Functions of PP2A

**Figure 5:**
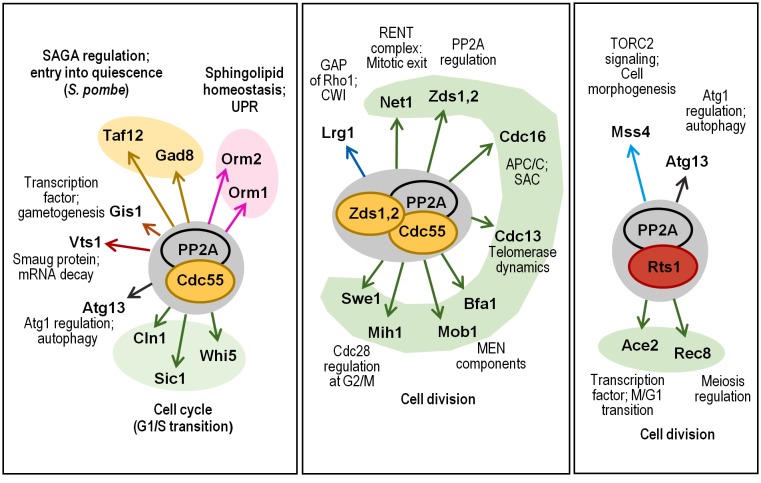
FIGURE 5: Multiple roles of PP2A in cellular functions. The cartoon shows the different combinations of PP2Ac with its regulatory subunits, and their role in different cell functions is depicted. Relevant substrates are also annotated and grouped with color codes denoting specific functions.

PP2A activity has been found as a regulator of multiple and essential cellular processes, such as the nutrient response, polarized growth and cell division [107]. We summarize here most of the recent advances on PP2A functions (**Figure 5**).

**Nutrient-related functions.** In fungi, as in other organisms, intracellular energy level controls cell growth. The antagonistic balance between the AMPK complex (SNF1 in fungi) and the Target of Rapamycin Complex 1 (TORC1) pathway senses the cellular energetic status. Both systems are conserved among eukaryotes and are susceptible of PP2A regulation, as recently reviewed [122, 123]).

PP2A participates in the nitrogen catabolite repression (NCR), triggered in yeast cells growing in the presence of a preferred source of nitrogen. In these conditions active TORC1 binds to and causes the phosphorylation of Tap42 that associates to PP2A (and to the PP2A-like phosphatase Sit4), decreasing PP2A activity and leading to the transcriptional repression of genes involved in the metabolism of less preferred nitrogen sources by preventing the activity of the downstream GATA transcription factors Gln3 and Gat1. The PP2A-Tap42 pathway regulates localization and function of the GATA transcription factors, modulating the NCR response. Inhibition of TORC1 by nitrogen depletion, addition of rapamycin or caffeine, dissociates from the Tap42–PP2A/PP2A-like complex from TORC1. This activates the phosphatase, required for dephosphorylation and nuclear localization, of the transcription factors [124]. Siw14, a tyrosine protein phosphatase necessary for the proper phosphorylation and localization of Gln3, has been identified as a negative regulator of PP2A in response to caffeine [125]. PP2A^Cdc55^ is also involved in the regulation of the nuclear accumulation and chromatin association of the environmental stress response transcription factors Msn2/4 [126].

Inhibition of the TORC1 complex by nutrient starvation induces the autophagic process, a catabolic response to nutrient deprivation. These conditions cause dephosphorylation of the TORC1 substrate Atg13, that binds to other Atg proteins and form the Atg1 kinase complex, required for autophagosome formation. It has recently been shown that both forms of PP2A (PP2A^Cdc55^ and PP2A^Rts1^) are needed for the dephosphorylation of Atg13 and induction of autophagy after the inactivation of TORC1 [127]. PP2A is also required for a “non-nitrogen starvation” induced form of autophagy, triggered by the lack of S-adenosylmethionine (SAM). In this form of autophagy PP2A itself acts as a sensor of the SAM concentrations, since the ability of the methyltransferase Ppm1 to methylate the catalytic subunit of PP2A is conditioned by the concentration of SAM, the substrate of the transferase [128].

Availability of nutrients modulates the cell size by a process that involves the PP2A^Rts1^ complex and its role in modulation of the expression of G1 cyclins [129]. Rts1 is also one of the ways to modulate the TORC2 signaling network via the dephosphorylation of the PI(4)P kinase Mss4 when cells are shifted to a poor carbon source. PP2A^Rts1^ seems to transmit not only nutrient-dependent but also ceramide-dependent signals as a feedback regulatory mechanism of the TORC2 network [130].

PP2A, together with PP1, are important regulators of mitosis in most eukaryotic organisms, as recently reviewed in [114]. In this regard, it is manifest that the roles of PP2A^Cdc55^ and PP2A^Rts1^ are not identical. PP2A^Cdc55^ regulates the G2/M transition and early mitotic exit. By contrast, PP2A^Rts1^ is mostly required for controlling cell size and spindle assembly checkpoints.

**Figure 6:**
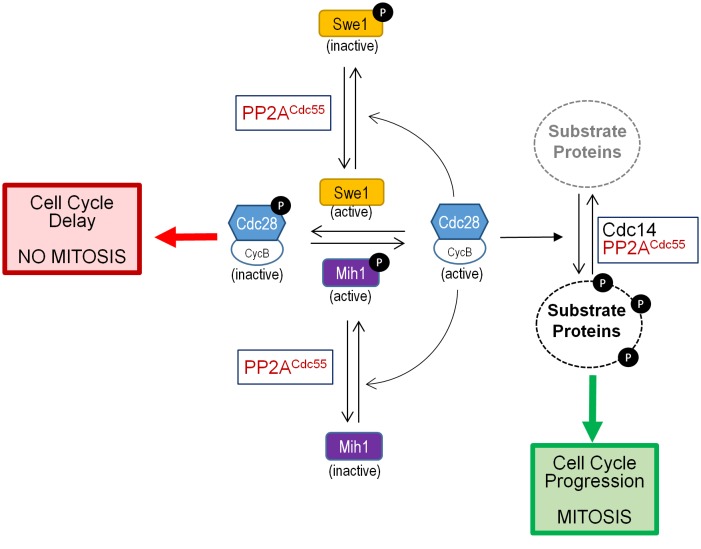
FIGURE 6: Role of PP2A^Cdc55^ in mitotic entry. The cartoon shows the participation of PP1A^Cdc55^ associated with Zds proteins (not shown) in the regulation of the phosphorylation state of the *S. cerevisiae* cyclin-dependent kinase Cdc28. See main text for details.

It is well established that entry into mitosis is triggered by phosphorylation of hundreds of proteins, substrates of the cyclin B-Cyclin-dependent kinase 1 (Cdc28 in budding yeast; Cdc2 in *S. pombe* and other organisms). It has been recognized during the last few years that progress into mitosis also requires the inhibition of PP2A^B55^, and that this is as important as the activation of Clb2-Cdc28 [131]. PP2A^B55^ regulates G2/M transition by dephosphorylating and activating the Cdk1 phosphatase (Mih1 in budding yeast and Cdc25 in *S. pombe*) during entry into mitosis by a conserved mechanism identified in both *S. cerevisiae* and *S. pombe*. The Cdk1 phosphatase, as the Cdk1 kinase (Swe1 in budding yeast, Wee1 in *S. pombe*) does, undergoes cycle-dependent changes in its phosphorylation state, being phosphorylated by its substrate Cdk1 [132] (**Figure 6**).

**Figure 7:**
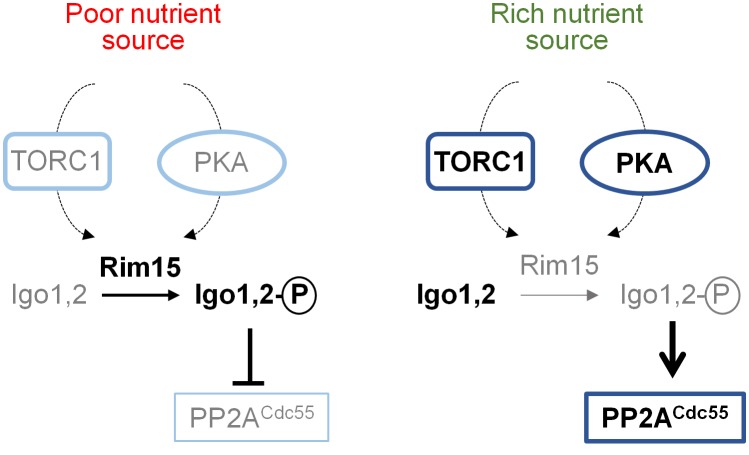
FIGURE 7: Regulation of PP2A^Cdc55^ functions by the Greatwall-endosul-fine pathway. The Rim15 protein kinase represents the Greatwall kinases in *S. cerevisiae*, whereas Igo1/2 are endosulfines. Prevalent species and processes under each specific condition (nutrient source) are denoted by bold font, whereas less active forms are in grey. See main text for details.

**Cell cycle-related functions (via Greatwall-ENSA pathway).** ENSA proteins negatively regulate PP2A^Cdc55^ functions in cell cycle in response to different cues [117]. In the yeast *S. cerevisiae* this family is represented by the pair of endosulfine-containing domain paralogs Igo1 and Igo2, although in other fungi a unique protein might exists. ENSA proteins are regulated by phosphorylation carried out by a member of the conserved Greatwall family of protein kinases (Rim15 in *S. cerevisiae* and Ppk18 in *S. pombe*) [133]. Activation of this Greatwall-ENSA module is initiated with the phosphorylation of the Igo proteins by the Greatwall protein kinase. Phosphorylated endosulfines are inhibitors of PP2A^Cdc55^ activity, as recently reviewed [134]. Inhibition of PP2A^Cdc55^ in the budding yeast delays cell cycle progression into mitosis, and the progression towards the exit from mitosis requires the dephosphorylation of Igo proteins in order to relieve the inhibition of PP2A^Cdc55^. Activation of Greatwall depends on nutrient availability and, in *S. cerevisiae*, requires the PKA and TORC1 kinases. Inhibition of TORC1 and PKA by low nutrient availability results in activation of Rim15 that, once translocated to the nucleus, phosphorylates Igo1/2 (**Figure 7**).

Modulation of the Greatwall-ENSA pathway in fission yeast controls the cell-cycle machinery coupling the nutritional environment to cell size. Thus, growth in the presence of a rich nitrogen source activates PP2A^Pab1^, which leads to subsequent activation of Wee1 that induces cell growth in G2 phase. On the contrary, inhibition of PP2A^Pab1^ under nitrogen deprivation releases the inhibitory effect of Cdc25 on Cyclin B-Cdc2, allowing shorter cells entry into mitosis because the shortened G2 phase [135–137].

**Figure 8:**
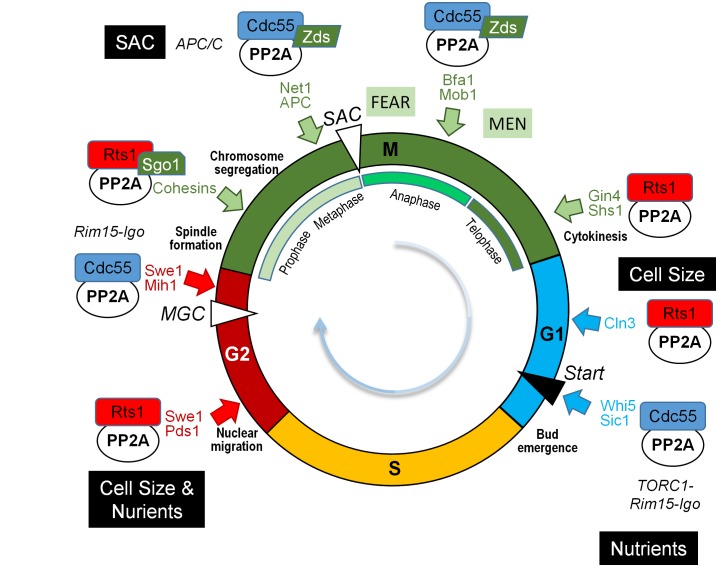
FIGURE 8: Multiple roles of PP2A in cell cycle. The cartoon shows the different combinations of PP2Ac and its regulatory subunits and their role in different points of the cell cycle. Relevant substrates are also annotated with the same color code than that the corresponding phase of the cell cycle. SAC, Spindle Assembly Checkpoint; MGC, Morphogenetic checkpoint.

Components of the CWI pathway are involved in the PP2A^Cdc55^-mediated regulation of cell cycle. Pkc1 has been identified as a protein kinase that plays a critical role in controlling phosphorylation of components of the PP2A^Cdc55^-ENSA complex, being the phosphorylation of Cdc55 by Pkc1 important for the dissociation of the ENSA protein from the complex [138]. In *S. cerevisiae* the Greatwall-ENSA-controlled PP2A^Cdc55^ coordinates nutrient availability with cell cycle progression by dephosphorylating Sic1, the inhibitor of the Cdc28-Clb complex, under TORC1 regulation, releasing the inhibitory effect of Slt2-phosphorylated Sic1 on G1/S transition (**Figure 8**). In fact, Slt2 and PP2A^Cdc55^ activities are reciprocally controlled by TORC1 [139].

Whi5, the repressor of the budding yeast G1-specific transcription, is dephosphorylated by PP2A^Cdc55^, thus antagonizing the phosphorylation exerted by Cdc28-Cln3. In early G1 phase, when PP2A^Cdc55^ is inhibited in response to low levels of Cdc28-Cln3, Whi5 is inactive, turning on the SBF transcription factor involved in cell size homeostasis. This allows transcription of the late Cln1 and Cln2 [136, 140]. PP2A^Cdc55^ stabilizes Cln1 and Cln2 levels by direct dephosphorylation of these cyclins, which prevents their SCF (E3 ubiquitin ligase complex)-dependent degradation until the end of G1 phase is reached [141].

Entry into gametogenesis and quiescence is also regulated by the Rim15-ENSA-PP2A^Cdc55^ module. Attenuation of the TORC1 and PKA pathways driven by limitation of nutrients activate Rim15 leading to inhibition of PP2A^Cdc55^. The transcription factor Gis1 has been identified as a substrate of PP2A^Cdc55^, and inhibition of the phosphatase promotes Gis1-driven transcription of specific nutrient-regulated genes to coordinate entry into quiescence [142]. In the same study, the mRNA-binding protein Vts1, a member of the Smaug family of proteins, was also identified as a PP2A^Cdc55^ substrate. Since Vts1 regulates mRNA stability interfering with the 5'-3' mRNA decay pathway, it could be possible that the Rim15-ENSA-PP2A^Cdc55^ module regulates not only transcription activation but also the protection from degradation of the newly expressed mRNAs [133, 142]. In response to nutrient limitation, the Rim15-ENSA-PP2A^Cdc55^ module is also required for pre-meiotic autophagy, but the mechanisms that regulate entry into gametogenesis are independent on the transcription factors Msn2, Msn4 and Gis1, involved in gametogenesis [143].

Recent phosphoproteomics studies suggest additional roles for PP2A^Cdc55^ in sensing several internal and environmental cues [144] being important for the septin ring dynamics and morphogenesis checkpoint (**Figure 8**). Thus, PP2A^Cdc55^ downregulates Swe1 after septin rings are properly organized, promoting mitotic entry, and it is also involved in the regulation of the septin ring disassembly process [107].

Mitotic exit requires the inactivation of the Greatwall kinase Rim15 to reactivate PP2A^Cdc55^ and initiate the dephosphorylation of the substrates previously phosphorylated by the Cdc28-cyclin B. This yeast pathway is part of a regulatory module that, although conserved during evolution, has evolved in other organisms to meet specific mitotic features [107, 137]. In *S. pombe* PP2A^Pab1^ determines the fate of the cell (sexual differentiation or cellular proliferation) depending on the availability of nutrients, by regulating the phosphorylation state of Taf12, a component of the SAGA complex. The phosphorylation state of Taf12 results from the balance of the TORC1-activated PP2A^Pab1^ and the TORC2-activated Gad8 protein kinase. Thus, under nitrogen-rich conditions TORC1-activated PP2A^Pab1^ dephosphorylates and inactivates Gad8 and also directly dephosphorylates Taf12, preventing the expression of mating genes [135, 145].

**Functions regulated by Zds proteins.** Zds proteins (Zds1 and Zds2 in *S. cerevisiae*) are important cell cycle regulators integrating multiple signals to control the activity of PP2A^Cdc55^ on different substrates by regulating the nucleocytoplasmic distribution of PP2A-Cdc55 along the cell cycle [146, 147]. Zds proteins undergo changes in its phosphorylation state along the cell cycle and, in fact, binding of Zds1 to PP2A^Cdc55^ is required for the continuous dephosphorylation of Zds1 [146]. It has been proposed that during mitosis, Zds proteins maintain PP2A^Cdc55^ in a cytoplasmic localization, excluding the phosphatase from performing nuclear functions such as the inactivation of nuclear Cdc28 at mitotic entry. Nuclear activation of both the APC-Cdc20 during metaphase/anaphase transition and of the Cdc14 phosphatase in the mitotic exit also require the nuclear exclusion of PP2A^Cdc55^ [147]. On the other hand, nuclear Zds1 also interacts with the Esp1 separase in a Cdc55-independent way, and recruits PP2A^Cdc55^ to the nucleolus, where Zds proteins down-regulate the phosphatase activity in early anaphase [148]. Zds proteins cannot be exclusively considered inhibitors of PP2A^Cdc55^ since, although they down-regulate PP2A^Cdc55^ during mitotic exit, these proteins promote PP2A^Cdc55^ functions for mitotic entry [146, 149]. Furthermore, Zds proteins are necessary for the cortical anchoring of PP2A^Cdc55^, which is important for the role of the phosphatase as a regulator of the GTP-binding protein Rho1 [150].

Binding of Zds proteins to PP2A^Cdc55^ is required to revert the inhibitory phosphorylation of Cdc28 at G2/M. There are evidences that this is accomplished by both inactivation of the Cdc28 phosphatase Mih1 and by dephosphorylation and activation of Swe1, the Cdc28 inactivating kinase. In fact, it has been proposed a model in which the Zds-PP2A^Cdc55^ module plays a negative role in the Swe1-driven G2/M morphogenesis checkpoint [116, 146] (**Figure 6**).

The PP2A^Cdc55^ module is also a regulator of the anaphase onset (**Figure 8**), which is triggered by activation of the Cdc20-dependent anaphase-promoting complex (APC^Cdc20^). Activation of the APC^Cdc20^ is required for the ubiquitin-dependent degradation of the securin Pds1, an inhibitor of the separase. Upon Pds1 degradation, active separase promotes sister chromatids segregation, by cleaving the cohesion complex, and triggers the FEAR (Cdc14 Early Anaphase Release) pathway that leads to the exclusion of Cdc14 phosphatase from the nucleolus. Release of Cdc14 from the nucleolus promotes its role as a key effector of mitotic exit (see [149] and references therein). Activation of the MEN (Mitotic Exit Network) occurs upon completion of chromosome segregation in late anaphase/telophase, where high levels of Cdc14 promote the destruction of the G2 cyclins and stabilization of the CDK inhibitor Sic1, thus inactivating the CDK and leading to mitotic exit. The PP2A^Cdc55^ module keeps dephosphorylated several subunits of the APC^Cdc20^ (such as Cdc27 and Cdc16) upon damaged spindle [147, 151], being the dephosphorylation of Cdc16 important for the adaptation to the metaphase arrest triggered by the SAC (Spindle Assembly Checkpoint) [152]. On SAC “satisfaction”, separase-driven down-regulation of Zds-PP2A^Cdc55^ alters the Cdc14 phosphatase nucleolar localization. This is caused by increased phosphorylation of Net1, a member of the FEAR complex, and by maintaining the phosphorylated form of Bfa1 and Bub2, members of the MEN regulatory network [153–156]. PP2A^Cdc55^ also participate in meiotic chromosome segregation since it is required for reductional chromosome segregation during achiasmate meiosis by a FEAR-independent mechanism [157, 158].

Removal of active telomerase from telomers at the G2/M transition is also regulated by PP2A^Cdc55^. The function of Cdc13, a ssDNA binding protein that binds to the telomerase subunit Est1 and interacts to Zds2, is regulated by phosphorylation, and it has been determined that Pph22-dependent dephosphorylation of Cdc13 negatively regulates the Cdc13-Est1 interaction and prevents telomerase recruitment during cell cycle progression [159].

The PP2A^Cdc55^–Zds1/2 complex has been identified as a Rho1 effector promoting, in the absence of stress, polarized growth and cell wall synthesis by one side, and inhibiting the CWI pathway by the other. This is accomplished by inhibition of the Rho1 GTPase-activating protein (GAP) Lrg1 and by stabilization of Sac7, another Rho1 GAP. Under cell wall stress the Slt2 MAPK pathway inhibits cortical PP2A^Cdc55^ forcing Rho1 to activate the CWI pathway for cell wall repair [150].

Cell division has different characteristics in the fission yeast, where PP2A^Pab1^ also plays important roles during cytokinesis, cell morphology and cell wall morphogenesis [160]. Thus, PP2A^Pab1^ regulates the SIN that, as the MEN in budding yeast, is required for the coordination of the onset of cytokinesis. In fission yeast, PP2A^Pab1^ negatively regulates the Rho1 GTPase, which is required for synthesis of cell wall and septum polymer [161]. The fission yeast orthologue of Zds proteins, Zds1, contributes to sexual differentiation, Ca^2+^ tolerance, maintenance of cell wall integrity, viability in the stationary phase and cell morphology. It remains to be determined if PP2A is involved in these processes in the fission yeast [162].

**Other functions of PP2A^Cdc55^.** PP2A^Cdc55^, one of the ceramide-activated PPases, is involved in the rapid inhibition of the signal triggered by heat stress that leads to sphingolipid biosynthesis through phosphorylation of Orm proteins. This PP2A^Cdc55^ function, that counteracts the early Ypk1 kinase-mediated phosphorylation of Orm proteins in response to the stress, ensures a transient sphingolipid production [163].

The lipolysis-dependent cell-cycle checkpoint, triggered by the absence of enough lipid precursors derived from triglycerides breakdown for the synthesis of sphingolipids, requires PP2A^Cdc55^. A model has been proposed where sphingolipids, identified long ago as effectors of PP2A^Cdc55^ function [164], are required to activate PP2A^Cdc55^, resulting in attenuation of Swe1 kinase phosphorylation and inhibitory effect on Cdc28 [165].

#### Functions of PP2A^Rts1^

PP2A roles related to correct chromosome segregation during cell division (in both meiosis and mitosis) and septin dynamics require the alternative B56 regulatory subunit (Rts1 in *S. cerevisiae*). The Rts1 subunit mediates the dephosphorylation of cohesin, protecting it from destruction, thus maintaining cohesion between centromeric regions but allowing the sister chromatids to resolve along the rest of the chromosome. PP2A^Rts1^ needs to associate to the Shugoshin protein (Sgo1 in *S. cerevisiae*; Sgo1 and Sgo2 in *S. pombe*). Sgo1 is a member of a functionally conserved family of centromere-related proteins, involved in the accurate chromosome segregation during cell division by sensing the lack of tension between kinetochores and spindle poles during the bipolar orientation [166]. Recent studies have pointed out that Sgo1 directly interacts to and is required for the recruitment of Rts1 to the centromere, configuring the PP2A^Rts1^-Sgo1 complex that will protect centromeric cohesin from premature removal [167]. PP2A^Rts1^ is necessary for the timely dissociation of Sgo1 from the pericentromere under spindle tension, as a part of a mechanism that ensures the proper sister chromatides bi-orientation of the mitotic spindle before the onset of anaphase. PP2A^Rts1^antagonizes the Bub1-driven phosphorylation of chromatin-associated substrates, which maintain Sgo1 bound to the pericentromere [167, 168]. In telophase, PP2A^Rts1^ induces the septin ring disassembly, a process mediated, at least in part, by Gin4- and Cla4-dependent phosphorylation of the Shs1 septin.

PP2A^Rts1^ also has important roles in meiosis I, protecting Rec8, a protein that contributes to maintain cohesion between centromeres of sister chromatids, from separase cleavage at the centromeres until meiosis II is reached. As in mitosis, PP2A^Rts1^ is recruited to the kinetochore in a Sgo1-dependent manner, and there the phosphatase counteracts the phosphorylation of Rec8 by the Hrr25 and Cdc7 protein kinases, while Rec8 remains phosphorylated throughout the rest of the chromosome arms (reviewed by [169]). Separation of dyad chromosomes during meiosis II needs the reactivation of separase at the centromers to cleave centromeric cohesin. In budding yeast, phosphorylation of centromeric cohesin is achieved by removing PP2A^Rts1^-Sgo1 from centromers in a process mediated by the Cdc20-dependent degradation of Sgo1 [170].

Moreover, PP2A^Rts1^ also controls cell cycle by regulating a number of transcription factors. Rts1 is important for the proper phosphorylation and localization of Ace2, a transcription factor required for expression in late mitosis and early G1 of genes involved in transport, ribosome biosynthesis, cell polarity, and septum destruction after cytokinesis, among other multiple functions [171]. Lack of Rts1 results in higher than normal presence of Ace2 in the mother cell nucleus, where it can activate *ASH1,* an inhibitor of the transcription of the *HO* endonuclease, leading to the blockage of *HO* expression in mother cells [172].

In the fission yeast, PP2A bound to the B56 subunits Par1 and Par2, inhibits the SIN in order to avoid multiple rounds of septation, probably by regulating the localization of the SIN kinase Cdc7 [173].

A functional interaction of PP2A^Rts1^ with the SAGA complex has been recently identified. *RTS1* has been described as high-copy number suppressor of several phenotypes caused by the deletion of *GCN5*, probably by restoring the low histone expression levels observed in the *gcn5* mutant strain [174]. Gcn5 is a member of the SAGA that targets several lysine residues of histones 2 and 3. Curiously, another high-copy suppressor of *gcn5* phenotypes found in the same study was *ZDS1* [174].

The multifaceted role of the various PP2A complexes during the different steps of the cell cycle is depicted in **Figure 8**.

#### Other functions of PP2A

PP2A plays a role in the decreased recruitment of Pol I to the 35S rDNA promoter induced by Cd^2+^, although the subunit required for this function is unknown [175].

A proteome-wide study performed in fission yeast has defined multiple biological roles where PP2A could be involved, including carbon and amino-acid metabolism, vitamin production, protein folding and the regulation of glycerol levels during osmotic stress response [176].

#### PP2A phosphatase as potential virulence determinant

PP2A, as a component of the STRIPAK-like complexes, plays important roles in growth, sexual development, and virulence in filamentous fungi, as previously reviewed [118]. The PP2A regulatory subunit ParA from *A. fumigatus*, indispensable for hyphal extension, conidiation and normal septation, seems not to be involved in virulence according to the results obtained with a *parA* mutant strain [177]. Both PP2A regulatory subunits of the pathogenic fungus *Aspergillus nidulans* play important roles in morphogenesis, conidiation, and self-fertilization, being involved in asexual and sexual development. In *S. pombe* both, PP2A^Par1^ and PP2A^Pab1^, inactivate the SIN pathway, which couples mitotic exit with cytokinesis. By contrast, in *A. nidulans* only PP2A^ParA^ is the negatively regulator of the SIN, counteracting the role of PabA during the septation process [178].

In *C. albicans*, septin Sep7 is dephosphorylated by Tpd3-bound Pph21. Dephosphorylated Sep7 is important for proper cell separation since *tpd3* mutant cells, defective in Sep7 dephosphorylation, are elongated, fail to separate cells, have a pseudohyphae-like morphology and are defective in hyphal growth. In agreement with these described phenotypes, *tpd3* mutant cells have greatly decreased their virulence, leading to the proposal of Tpd3 as a target for antifungal drugs. Collectively, these results sustain a role of PP2A in filamentous fungi pathogeny.

### PPH3

The *S. cerevisiae* gene *PPH3* codes for a type 2A-related phosphatase catalytic subunit, showing 52% and 58% identity with Pph21 and Pph22, respectively (**Figure 4**). Although Pph3 is not an essential protein, the gene was found required for survival in the absence of *PPH21* and *PPH22* [106], thus suggesting partially overlapping functions with PP2A enzymes. As it occurs with PP2A, Pph3 enzymes are present in all fungal species analyzed. However, early enzymatic characterization and phenotypic analyses already hinted that Pph3 function(s) differs from that of PP2A or Sit4 enzymes (see [46] and references therein). In addition, and contrary to PP2A enzymes, Pph3 is not methylated by the Ppm1 methyltransferase [179]. Pph3 has been often found associated with two other proteins, Psy2 and Psy4, which would be acting as regulatory components of a phosphatase complex that has been maintained through evolution and is also found in humans [180, 181]. The human counterparts of Pph3, Psy2, and Psy4 would be PP4c, R3, and R2, respectively. However, some differences exist, since while association of the human R3 subunit appears to depend on preassembly of Pp4c and R2, yeast Pph3 and Psy2 form a stable complex even in the absence of Psy4. Pph3 is able to interact with the Peptidyl-prolyl cis/trans-isomerase Rrd1/Ypa1 that activates phosphotyrosyl phosphatase activity in PP2A and PP2A-like enzymes [113], and such interaction is relevant for certain cellular functions of the phosphatase [182–184].

The Pph3 phosphatase has been related to diverse functions. Early work linked Pph3 to a role in the TOR signaling pathway that regulates NCR through the GATA-type transcription factor Gln3, in contrast with the previously reported involvement of the Tap42-Sit4 complex (see [46] for references). However, further work provided data suggesting a minimal influence of Pph3 on Gln3 regulation compared with that of Sit4 [185]. More recently, it has been proposed the requirement of Pph3 activity in dephosphorylating Maf1, the major repressor of RNA polymerase III (Pol III) transcription, in response to nutrient deprivation (thus counteracting the function of Tor and PKA kinases), or to diverse stresses [186]. The role of Pph3 in the dephosphorylation of Maf1 would involve the scaffold Psy2, as well as Rrd1 and Tip41 (a Tap42-interacting protein, see above). Pph3 is also connected to the response to glucose starvation, and it has been proposed that the Pph3-Psy2 complex counteract the major glucose-responsive kinase PKA by dephosphorylating the putative PKA sites in Mth1, a protein needed for efficient repression of *HXT* glucose transporters upon glucose deprivation [187]. Targeting of Mth1 would be accomplished through direct binding of the EVH1 domain of the Psy2 regulatory subunit to the polyPro motif of Mth1.

On the other hand, Pph3 has been related to the response to DNA damage. Initiation of the response to DNA damage involves the sequential activation of the Mec1 and Rad53 kinases, finally affecting the phosphorylation state of numerous downstream proteins. The role of Pph3 counteracting this phosphorylation cascade is multifaceted. For instance, Pph3 was recognized as a Rad53 phosphatase, forming a complex with Psy2 that binds and dephosphorylates activated Rad53 [188], thus allowing resume of cell cycle progression once the problems have been solved. Lack of Pph3-Psy2 caused delayed Rad53 dephosphorylation and resumption of DNA synthesis during recovery from DNA damage, due to failure to restart stalled replication forks [188]. These authors reported that the role of Pph3/Psy2 seems to be required for cellular responses to the DNA-damaging agent methyl methanesulfonate but not the DNA replication inhibitor hydroxyurea (HU). Pph3, however, is not the only phosphatase participating in Rad53 dephosphorylation, and a role for Ptc2/3 and Glc7 phosphatases has been also reported [189–192]. More recent studies suggest that Pph3 mainly acts on pools of active Rad53 that have diffused from the site where DNA lesions occur [193].

Pph3 also impacts on DNA damage checkpoint through regulation of the Mec1 kinase, which phosphorylates and activates Rad53. It has been shown that Mec1-Ddc2 and Pph3-Psy2 physically interact in a DNA damage-independent manner and that Mec1-Ddc2 and Pph3 co-regulate many Mec1-dependent phosphorylation targets in response to HU stress, such as Rad53 and Histone 2A (H2A). In addition, Ser1991 phosphorylation in Mec1 was regulated in a Pph3-dependent manner [194]. Finally, targets downstream Rad53 are also affected by Pph3. H2A is phosphorylated at Ser129 (giving rise to the γH2AX variant) in a Mec1/Tel1 dependent manner in response to either DNA double-strand breaks (DSBs) or stalled replication. It was shown that Pph3, in conjunction with both Psy2 and Psy4, is required to dephosphorylate γH2AX [195]. This ability was subsequently confirmed for the human PP4 homolog [196].

Pph3 would participate in the two independent pathways governing the mechanisms for DSB repair: 1) homologous recombination, in which it would be redundant with Ptc2/3 [182]; and 2) Non-Homologous End Joining [197]. Cells deficient in Pph3 activity show coordinate blockage at early stages of both crossover repair and homology-independent pairing of centromeres. Such defect was linked to persistent hyperphosphorylation of Zip1, a filament protein of the synaptonemal complex and required for normal levels of meiotic recombination and pairing between homologous chromosomes [198]. Thus, it was proposed that Pph3, in association with Psy2, would counteract Mec1-induced phosphorylation of Zip1 at Ser75, and promote chromosomal pairing. The participation of Pph3 in defining a novel intranuclear quality control compartment (INQ) that sequesters misfolded, ubiquitylated and sumoylated proteins in response to genotoxic stress has been recently proposed [199].

The role of Pph3 in the response to DNA damage, in particular its relationship with Rad53 dephosphorylation, has been also investigated in the pathogenic yeast *C. albicans*. It was shown that Pph3 and Psy2 are required for the dephosphorylation of Rad53, but not γH2AX, and that deletion of the corresponding genes yielded strong filamentous growth under genotoxic stress [200]. As in *S. cerevisiae*, Pph3/Psy2 are required for the response to DNA damage caused by methyl methanesulfonate but not by HU [201]. More recent work has revealed Rad53 Ser residues 351, 461 and 477 as likely targets for Pph3-mediated dephosphorylation [202]. Pph3 is also responsible for dephosphorylation of Rfa2, a subunit of the replication protein A complex that is phosphorylated by Mec1 and the cyclin-dependent kinase Clb2-Cdc28 in response to the genotoxic insult [203].

### The SIT4 (PPH1) phosphatase

**Figure 9:**
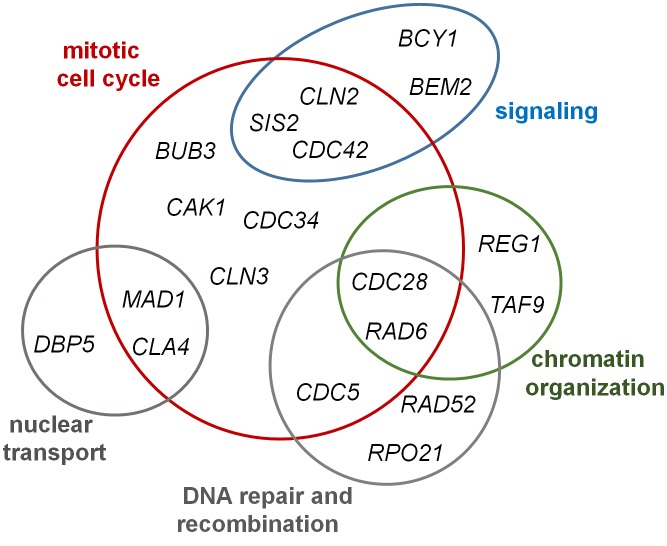
FIGURE 9: Genes displaying synthetic lethality with the *sit4* mutation. Venn Diagram showing GO categories for genes displaying synthetic lethality with the *sit4* mutation. The list was collected from SGD and analyzed with the Gene Ontology Slim Mapper tool. Only *SSD1, RPB2* and *PRE1* interactions are not included.

The *S. cerevisiae* gene *SIT4* (also known as *PPH1*) encodes a type 2A-related protein phosphatase of 311 residues (**Figure 4**) that was initially cloned in a screening for restoration of *HIS4* expression in strains lacking Bas1, Bas2 and Gcn4. Two years later it was found necessary for progression during the G1/S cell cycle transition (see [46] and references therein). Sit4 is required for expression of *CLN1* and *CLN2* G1-cyclins, as well as of the transcription factor *SWI4*, and cells lacking the phosphatase do show defects in bud emergence [73, 204]. Deletion of *SIT4* in some genetic backgrounds (cells lacking *SSD1* or harboring defective *ssd1*-d alleles) is lethal, whereas in other backgrounds cells are viable but display a noticeable slow-growth phenotype. The relevant role of Sit4 in cell cycle regulation is highlighted by the observation that, in addition to *ssd1*, the *sit4* mutation is synthetically lethal with more than 20 genes, of which almost half are related to the mitotic cell cycle (**Figure 9**). The Sit4 protein is conserved throughout evolution in eukaryotes. Indeed, overexpression of human PP6 or Drosophila PPV reverts the slow-growth defect of a *sit4* mutant, indicating that these proteins are functional homologs [205, 206].

#### Regulation

Sit4 associates with a number of proteins. Luke and coworkers [207] reported the cell cycle-dependent interaction of Sit4 with several proteins, Sap155, Sap185, Sap190 and Sap4, collectively named as SAPs (Sit4-associated proteins) and demonstrated that loss of all four SAP was phenotypically equivalent to the loss of Sit4 in term of delayed G_1_ to S phase cell cycle progression, slower growth, and budding defects. On the basis of amino acid sequence and functional behavior, SAPs could be classified into two groups: Sap185 and Sap190 are more similar to each other than to Sap155 and Sap4 [207]. Current evidence suggests that the SAP proteins positively regulate Sit4 phosphatase activity, and probably also its substrate specificity [207–210]. SAP proteins could be, up to some extent, conserved through evolution, since it has been shown that three putative human homologs (PP6R proteins) physically interact with Sit4 and provide some, but not all, Sit4-dependent SAP functions when expressed in budding yeast [211]. Sit4 can form a different heterotrimeric complex with Tpd3 and Cdc55 (the typical A and B PP2A subunits), giving rise to a ceramide-activated protein phosphatase [164]. A link between ceramide homeostasis and the UPR through Sit4 has been reported [212].

Sit4 also associates, in a SAP-independent fashion, to Tap42 [213], an essential protein that is involved in one of the branches of TORC1-mediated signaling. When preferred nitrogen sources are available, active TORC1 phosphorylates Tap42, thus promoting the interaction between Tap42 and Sit4 (as well as with other type 2A PPases) and restricting access of the PPases to their cellular targets (see [214] and references therein). Upon starvation of nitrogen, treatment with rapamycin (a TORC1 inhibitor), or in the presence of certain stresses, the Tap42-PPase complex dissociates and diverse cellular targets are dephosphorylated, such as Gln3 and Gat1, which translocate to the nucleus and trigger the transcription of genes involved in the metabolism of non-preferred nitrogen sources [214–216]. Tip41 has been described either to collaborate or oppose to TORC1 signaling through Tap42-Sit4 [111, 217]. It must be noted that a different brand of phosphatase, Ptc1, has been proposed to act upstream of Sit4 in TORC1 signaling and influence Tip41 stability and hence Tip41-Tip2 association [218]. It has been also shown that Sit4 mediates TORC1 signaling to maintain nuclear localization and stability of the transcription factor Stp1 and hence promoting amino acid uptake [219]. More recently, a role for Tap42-Sit4 in controlling site-specific acetylation of histone H3 and H4 N-terminal tails, thus controlling epigenetic traits, has been proposed [220].

Sit4 also interacts physically and is regulated by the phosphotyrosyl phosphatase activators (PTPA) Ncs1/Rrd1 and Noh1/Rrd2, also known as Ypa1 and Ypa2 [113, 221, 222], which also regulate other type 2A PPases. It has been demonstrated that Rrd1 can be a component of the Tap42-Sit4 complex, and that rapamycin promotes the release of the PTPA-Sit4 active complex [223].

#### Functions

In addition to its involvement in cell cycle progression and TORC1 pathway signaling, mentioned above, Sit4 regulates a broad range of biological processes. For instance, the phosphatase plays a role in the CWI pathway, since deletion of *SIT4* enhances both basal and heat-induced phosphorylation level of the Slt2 MAP kinase, and the phosphatase appears involved in rapamycin-mediated induction of Slt2 [209, 224]. It was shown that Sap185 and Sap190 function together with Sit4 to provide an essential role in the absence of Bem2 [207], a GTPase activating protein that down-regulates Rho1. However, the additive effect of the *sit4* and *bem2* mutation on Slt2 does not support the notion of Sit4 being a regulator for Bem2 and suggest an independent role for Sit4 and Bem2 on Slt2 regulation [224].

A role for Sit4 in monovalent cation tolerance and pH homeostasis was proposed by Masuda and coworkers, on the basis that overexpression of the phosphatase conferred lithium tolerance in galactose medium but, in contrast to the mutation of Ppz1, this effect did not affect the expression of the *ENA1* ATPase gene [225]. It was also observed that Sit4-overexpressing cells maintain a more alkaline intracellular pH than wild type cells. Interestingly, it has been very recently shown [226] that rapamycin inhibits the H^+^-ATPase Pma1 in a way that depends on Sit4. Since the *sit4* mutant exhibits low Pma1 activity, the authors propose a mechanism by which TORC1 activates Sit4 and the phosphatase, directly or indirectly, activates Pma1. This reported effect of Sit4 on Pma1-dependent H^+^ efflux might explain the changes in intracellular pH described by Masuda and coworkers. A role for Sit4 has also been proposed in K^+^ homeostasis [227], likely through regulation of the Nha1 H^+^/Na^+^,K^+^ antiporter. In this case, Sit4-dependent opposite effects of Sap155 and Sap185 overexpression were observed, being S*AP155* and *SAP185* negative and positive modulators of K^+^ efflux, respectively. However, K^+^ efflux was not affected by the mutation of *SIT4* [227]. Interestingly, *NHA1*, encoding the yeast H^+^/Na^+^,K^+^ antiporter, was found as a high-copy suppressor of the synthetic lethality of the *sit4* and *hal3/sis2* mutations [85]. However, this effect is likely unrelated to the role of Nha1 in maintaining K^+^ and pH homeostasis, as deduced from mutagenesis analysis of ScNha1 and heterologous expression of the *C. albicans* antiporter [228, 229].

Sit4 plays a role on lipid metabolism. For instance, mutants in *sit4* and *sap190* were catalogued as low-lipid droplet content strains, whereas the content in *sap185* cells was higher than normal [230]. In addition, it has been shown that *sit4* deletion mutants have decreased ceramide levels, display resistance to exogenous ceramides and phytosphingosine, and show a shift towards non-hydroxylated forms of long chain bases and sphingolipids, suggesting that Sit4 could regulate hydroxylase (*SUR2*) or ceramide synthase, without involvement of the SAPs regulators [231]. A link between the TOR pathway and lipid droplets mediated by Sit4 and Tap42 and requiring the downstream TORC1-controlled transcriptional activators Gln3, Gat1, Rtg1, and Rtg3 has been reported [232].

The Sit4-Sap190 and/or Sit4-Sap185 complexes are also required for normal Elongator activity [233]. A major function of the Elongator complex in yeast (composed of Elp1 to Elp6) is the formation of certain modifications at the tRNA anticodon, and it is known that *sit4* and *elp1–elp6* mutants display identical tRNA modification defects (see [234] for a review). The role of Sit4 seems to antagonize the phosphorylation of the largest Elongator subunit Elp1 by the Hrr25 kinase. Recent work has proved that the role of Sit4 on lipid droplet synthesis is independent of its function on Elongator-dependent tRNA modification [235].

Carbohydrate metabolism is also affected by Sit4 activity. It has been proposed that lack of *SIT4* causes rewiring of carbohydrate metabolism, with entry in a futile cycle of glycogen synthesis and degradation, down-regulation of fermentation, overexpression of genes that are normally activated by glucose deprivation, and activation of respiration [236]. The reduced fermentative capacity of *sit4* cells has been attributed to a decrease in pyruvate decarboxylase activity [237]. In cells actively growing in the presence of abundant glucose, the Mig1 repressor and Hxk2 are dephosphorylated and transferred into the nucleus where this complex exerts a repressor effect on expression of genes required for growth on non-fermentable carbon sources. It has been found that in the absence of Sit4, the Snf1 kinase is activated and then phosphorylates the Mig1 repressor, which leads to its inactivation [25, 238]. Sit4 also influences catabolite repression in a Snf1-independent fashion, since lack of the phosphatase promotes the degradation of the Mig1 repressor [239] Furthermore, hyperphosphorylation of Hxk2 observed in *sit4* mutants prevents the formation of the Mig1-Hxk2 complex. Free Mig1 is then phosphorylated at Ser311 by Snf1 promoting export of the repressor into the cytosol. This further contributes to interfere with normal glucose repression [240, 241]. Sit4 is also involved in the link between Snf1 and protein translation since, whereas in histidine starved cells Snf1 promotes the formation of phospho-eIF2α by activating the Gcn2 kinase, when cells are shifted from glucose to galactose Snf1 counteracts the likely direct Glc7 and Sit4 phosphatase activity on phospho-eIF2α [242].

As mentioned, respiration is derepressed in *sit4* cells grown in glucose medium. Since these mutants are unable to grow under anaerobic conditions, mitochondrial respiration becomes essential for their viability. Mitochondria are a major source for reactive oxygen species and play key roles in oxidative stress resistance and chronological lifespan. In agreement with the proposed role of Sit4 as a negative regulator of mitochondrial function, *sit4* cells show some protection from defects associated with mitochondrial DNA damage [243], and increased chronological lifespan [244]. It has been recently found that Hxk2 is hyperphosphorylated in *sit4*-deficient cells by a Snf1-independent mechanism, and that mutation of Ser15 in Hxk2 to Ala suppressed diverse phenotypes associated with the deletion of *SIT4*, such as transition defects at G1 phase, derepression of mitochondrial respiration, tolerance to H_2_O_2_ and lifespan extension [245]. Very recently, involvement of Sit4 in the post-translational regulation of nine mitochondrial proteins has been reported. In the case of the ATP synthase β subunit Atp2, it was proposed that Sit4-mediated dephosphorylation of Atp2 at T124 and T317 downregulates Atp2, together with ATP synthase and mitochondrial function [246].

### PPG1

The *S. cerevisiae PPG1* gene encodes a 368 residues protein with some similarity to type 2A phosphatases (**Figure 4**). However, it features an internal insertion of ten residues (from amino acids 205-215) and a C-terminal extension of ~50 residues ending with the highly conserved DYFL sequence characteristic of type 2A phosphatases [247]. This phosphatase is also found in other fungi, but seems absent in human or plants, where the closest sequences are that of PP4 or PPX phosphatases. Ppg1 has been found to interact with Tap42 [248] and Tip41 [249], which are proteins involved in the TORC1 signaling pathway and also able to interact with other type-2A phosphatases. In addition, interactions of Ppg1 with canonical PP2Ac regulatory subunits, such as Cdc55 and Tpd3 [51] or Rrr1/Ypa1 [113], have been described.

Initial characterization of *ppg1* mutants revealed decreased glycogen accumulation that could be attributed to higher levels of glycogen phosphorylase **a**, in addition to lower amounts of total glycogen synthase activity [247]. More recently, it was found that *ppg1* mutants were tolerant to ethanol and heat [250] as well as sensitive to Congo Red [251], a compound that interferes with normal cell wall synthesis. The latter phenotype fits with the finding that, in *C. neoformans, ppg1* mutants are also sensitive to cell wall inhibitors, such as Congo Red or Calcofluor White (CFW) [252]. It must be noted that although in this paper the Cryptoccocus Ppg1 protein is defined as a "Sit4 homologue", Blastp analysis of the reported GenBank entry (XP_571206) shows higher identity to *S. cerevisiae* Ppg1 than to Sit4.

Given its interactions with Tap42 and Tip41, it is conceivable that Ppg1 might act downstream the TOR pathway. Indeed, mutations in *PPG1* were found to suppress TORC2 deficiency [253], and it was proposed that the phosphatase might collaborate with the FAR complex in the control of actin-based cell polarity. As mentioned above, the FAR complex is composed of Far3 and Far7-11 and components of this complex interact functionally and physically with PP2A phosphatases [119]. Very recently, Ppg1 has been shown to interact with members of the FAR complex within the context of dephosphorylation of the mitophagy receptor Atg32, and it has been proposed that Ppg1 and the FAR complex cooperatively counteract casein kinase 2-mediated phosphorylation of Atg32 to prevent excessive mitophagy [254].

Studies in *C. albicans* using the systematic deletion mutant library have shown that Ppg1 is important for virulence [255]. In addition, Ppg1 was found necessary for filament extension, invasion, and virulence in a mouse model of systemic candidiasis. It was also proposed that Ppg1 controls *C. albicans* filamentation via the PKA signaling pathway, and that is also important for downregulation of *NRG1*, encoding a transcriptional repressor required for filamentous growth [256].

## PP2B (PP3, CALCINEURIN) AND PP2B-RELATED ENZYMES

**Figure 10:**
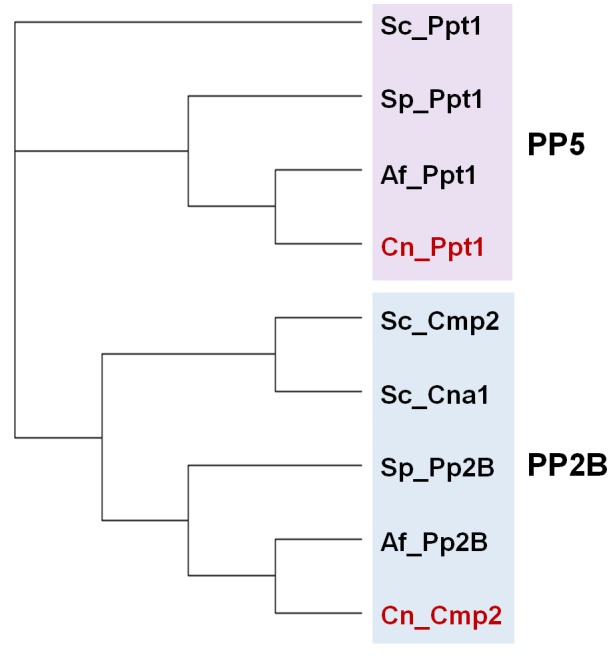
FIGURE 10: Phylogenetic relationship of PP2B and PP5 (Ppt1) phosphatases from various fungal species. Protein sequences correspond to organisms described in Figure 2. The analysis was performed as described in Figure 1.

This family comprises two branches: calcineurin (PP3) and PP5. Both kind of enzymes are widely distributed among eukaryotes, including fungi (**Figure 10**).

### Calcineurin

**Figure 11:**
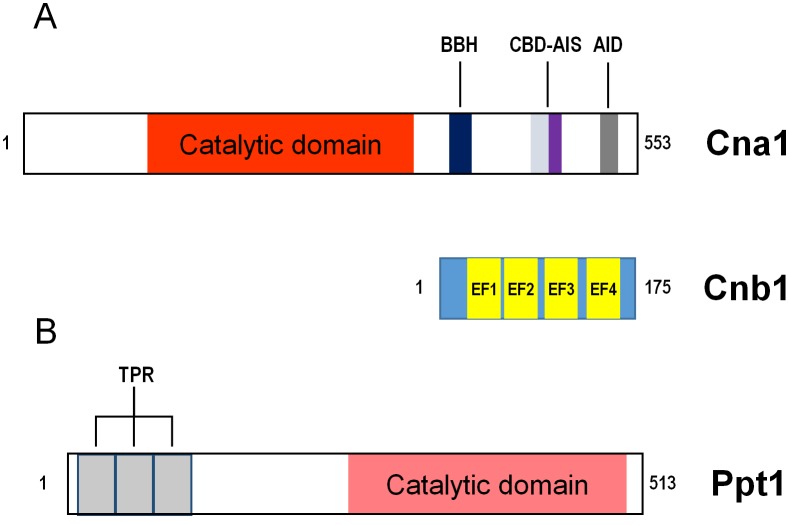
FIGURE 11: Schematic depiction of the structure and functional features of the catalytic (Cna1) and regulatory (Cnb1) subunits of calcineurin (A), and of Ppt1 (B). BBH, calcineurin B binding Helix; CBD, Calmodulin Binding Domain; AIS, AutoInhibitory Signal; AID, AutoInhibitory Domain. EF1-4, EF hand Ca^+2^ binding domains. TPR, tetratricopeptide repeats. The number of TRP repeats shown are according to SMART analysis. The number of residues is indicated on the right of each figure. See main text for details.

Calcineurin is a conserved Ser/Thr protein phosphatase regulated by calcium/calmodulin. In budding yeast calcineurin is a heterodimer, made out of one of two redundant catalytic subunits (encoded by the *CNA1* and *CNA2* genes), plus a regulatory subunit, encoded by *CNB1* [257]. The *CNA1* gene product has 553 residues, whereas the protein encoded by *CNA2* is somewhat larger (604 amino acids). The phosphatase domain is located at the N-terminal half of the so called calcineurin A (CNA) polypeptide, and is followed by a regulatory subunit binding helix (BBH), a calmodulin-binding domain (CBD), and a C-terminal autoinhibitory domain (AID) that blocks the catalytic activity [258] (**Figure 11**).

Cnb1 is a relatively small protein (175 residues) that contains four EF hand calcium binding domains (EF1-4, **Figure 11**). *In vitro* analysis of recombinant mammalian calcineurin allowed to propose that EF3 and EF4 have a structural role and constitutively bind Ca^2+^ in the cells, whereas calcium binding to EF1 and EF2 facilitate responsiveness to changes in intracellular Ca^+2^ because such interaction results in conformational changes in CNB that trigger partial activation of the phosphatase catalytic subunit. Additional binding of Ca^2+^/calmodulin to CNA would cause dissociation of the so called autoinhibitory signal (AIS) from the LxVP docking pocket and reorientation of the C-terminal AID with respect the catalytic site allowing full enzyme activation [259]. Recent work using yeast calcineurin [260] demonstrated that, as previously reported for mammalian calcineurin, EF4 is largely dispensable for calcineurin activity in response to intracellular Ca^2+^ signals, whereas EF1, EF2 and EF3 each contribute to calcineurin activation, independently of previously known conformational changes induced upon Ca^2+^/calmodulin binding. The regulatory properties of Cnb1 are affected by myristoylation of the N-terminal Gly by the Nmt1 *N*-myristoyltransferase, since mutation of this Gly or expression of the *ts* allele *nmt1-181* increases calcineurin activity [261]. Therefore, Cnb1 myristoylation functions to limit calcineurin signaling.

Calcineurin recognizes its substrates and interacting proteins via conserved short, degenerate docking motifs that are distinct from the sites of dephosphorylation. One of such motifs, known as PxIxIT sequence (with a [PG]xΦxΦζ consensus) has been identified in several calcineurin substrates in *S. cerevisiae*, such as Crz1, Rcn1, Slm1, Sml2, or Hph1 (see, [262] and references therein). These interactions, which are of relatively low affinity, occur in CNA at a groove distal from the catalytic center and independently of the phosphatase activation state. In addition, a secondary contact can be made through the LxVP motif, also found in some substrates such as Rcn1, which is thought to mediate the orientation of substrates during dephosphorylation, and therefore are only relevant when calcineurin is active. More recently, a combination of phosphoproteomics and bioinformatic approaches has expanded the list of yeast calcineurin candidate substrates, including the kinase Elm1 (acting upstream the Snf1 kinase) or Dig2, involved in pheromone signaling [263] (see below).

Calcineurin was recognized long ago as the target for the immunosuppressive drugs cyclosporin A (a cyclic peptide) and tacrolimus (FK506, a macrolide). These drugs form complexes with cyclophilin (Cpr1) and the FK506 Binding Protein (Fpr1 or FKBP12, a ubiquitously expressed peptidyl-prolyl isomerase), respectively, and these complexes are responsible for calcineurin inhibition. The resolution of the crystal structures of calcineurin and its complexes with FKBP12-FK506 and cyclophilin-cyclosporin allowed the identification of a few common residues in calcineurin required for recognition of the complexes [264]. It has been documented that the LxVP motif is necessary for interaction with the immunosuppressant-immunophilin complexes, raising the notion that they inhibit calcineurin by interfering with substrate recognition [265]. Recently, a mechanism of self-substrate regulation unique to the *A. fumigatus* and *C. albicans* FKBP12 proteins has been proposed [266].

RCANs (Regulators of calcineurin) are a family of proteins known to modulate calcineurin activity. Although also found in humans, RCANs were first identified in yeast because their ability to interact with and inhibit calcineurin upon overexpression. Indeed, signaling via calmodulin, calcineurin, and Crz1 (the transcription factor downstream calcineurin, see below) induced Rcn1 expression, suggesting that Rcn1 works as an endogenous feedback inhibitor of calcineurin [267]. However, there has been some controversy regarding the physiological roles of these regulators, since in the same work it was shown that loss of *RCN1* in yeast also gave rise to decreased calcineurin signaling. A positive role of Rcn1 (which can be extended to mammalian RCANs) was reinforced by the finding that the stimulatory effect of yeast Rcn1 involves its phosphorylation at a conserved serine residue by Mck1, a member of the GSK-3 family of protein kinases. This allowed postulating that Rcn1 might act as activator or inhibitor of calcineurin depending of its phosphorylation state [268]. A subsequent comparative study identified conserved docking motifs that were necessary for inhibition of calcineurin signaling, whereas several additional motifs in RCANs (such as the GSK-3 phosphorylation site) were specifically required for stimulatory and not for inhibitory effects. The authors suggested that RCANs may function primarily as chaperones for calcineurin biosynthesis or recycling [269].

#### Function

In budding yeast calcium is a common second messenger for diverse stimuli, such as exposure to mating pheromones, high salt or osmolarity, endoplasmic reticulum stress, and others (see [270, 271] and references therein), although the timing of the calcium response varies greatly (from seconds in response to alkalinization [272] to nearly one hour upon stimulation with mating pheromone [273]). Calcineurin is a major cellular target for the calcium signal, as exemplified by the observation that nearly 50% of the gene deletions that lead to calcium sensitivity are suppressed by inhibition of calcineurin [274].

Activation of calcineurin provokes multiple changes in the yeast cells. Many of these changes are promoted by the calcineurin-mediated dephosphorylation of the zinc-finger transcription factor Crz1/Tcn1/Hal8 (from now on, Crz1). Upon binding to its PxIxIT-like motifs, calcineurin dephosphorylates Crz1 at various sites and this promotes fast entry of Crz1 into the nucleus, assisted by Nmd5 (see [257] and references therein). Binding of Crz1 to gene promoters occurs at somewhat degenerate consensus sequence, named CDRE (Calcineurin-Dependent Response element) that exhibits a constant GCC core. Such CDRE was defined by Yoshimoto and coworkers as [TG(A/C)GCCNC] or [CAGCCTC], depending on the methodology used [275], or as A/CGCCNC by means of Protein Binding Microarray technology [276]. A more recent study using ChIP-Seq technology identified 152 intergenic regions recruiting Crz1 upon alkaline stress (the vast majority between 1 and 5 min upon stress onset), and confirmed the prevalence of the A/CGCCNC motif for Crz1 binding [277]. These authors also showed that the presence of the C at the 3' position of the CDRE was more frequent in promoters showing strong Crz1 recruitment. It has been demonstrated that, in response to external calcium, Crz1 shows pulsatile localization dynamics, with stochastic short burst ( ~2 min) of nuclear localization [278, 279].

The set of genes induced upon calcineurin-mediated activation of Crz1 encode proteins controlling diverse cellular functions. Monovalent cation homeostasis is affected by the absence of calcineurin in several ways. In addition to a possible direct effect of calcineurin on the Trk potassium transporters switch from low to high affinity transport [58], calcineurin/Crz1 activates expression of *HAL5* [280], encoding a kinase important for stabilization of Trk1/2 at the plasma membrane. Sodium efflux under cation stress is greatly influenced by Crz1, which plays a major role in the control of *ENA1* expression [281–283]. Activation of calcineurin influences calcium homeostasis and results in Crz1-mediated increase in the expression of *PMC1* and *PMR1* (encoding Ca^2+^ ATPases in the vacuole and the Golgi apparatus, respectively). Calcineurin also negatively regulates the vacuolar Ca^2+^/H^+^ exchanger Vcx1 and influences Ca^2+^ influx through the plasma membrane Cch1/Mid1 calcium channels, likely by controlling the phosphorylation state of Cch1 (see [273] and references therein). Additional substrates of calcineurin, independently of its role on Crz1, are Hph1 and the Slm1/Slm2 proteins (see below). Hph1 and Hph2 are homologous proteins with overlapping functions, required for normal tolerance to saline, alkaline pH, and cell wall stress [284]. Calcineurin-mediated dephosphorylation positively modulates Hph1. More recently it has been described [285] that Hph1 (and Hph2) act with the Sec63/Sec62 complex and that defects in this complex results in destabilization of Vph1, a subunit of the vacuolar proton ATPase (V-ATPase).

Calcineurin plays a role in the regulation of the biosynthesis of sphingolipids, and this involves the ancillary TORC2 subunits Slm1 and Slm2 [286], which were initially described as direct substrates for calcineurin [287]. It has been proposed that calcineurin negatively regulates the sphingolipid pathway at the level of ceramide synthesis, and this is largely due to the direct dephosphorylation of the ceramide synthase subunits Lac1 and Lag1 [288]. Such dephosphorylation would counteract the positive effect of phosphorylation due to TORC2-Ypk1 signaling.

Quite often, those conditions that activates the Slt2-mediated CWI pathway also triggers influx of calcium and activation of calcineurin (note that Mid1 has been proposed to be a mechanosensitive channel). Both the CWI pathway and the calcineurin pathways are important in response to cell wall stress, and when one of them becomes non-functional, the other becomes essential [289]. More recently, it has been shown that Pkc1-Slt2 and calcineurin pathways cooperate to allow cell survival under compressive mechanical stress [290]. Knr4, an intrinsically disordered protein, has been proposed to serve as connecting node between these two signal transmission pathways [291].

Calcineurin plays a significant role in the adaptation to nutrient availability. In response to glucose addition, yeast cells raise a calcium signal that occurs through two different influx pathways: Mid1/Cch1 and the GIC (for Glucose Induced Calcium) system, and that involves IP_3_ as second messenger [292, 293]. This signal can stimulate calcineurin and results in the up-regulation of the expression of diverse genes encoding carbohydrate transporters and metabolizing enzymes. Such activation is sufficient to allow growth under glucose limitation even in the absence of the Snf1 kinase [294]. Likewise, in response to excess amino acids, dephosphorylation of the α-arrestin trafficking adaptor, Aly1/ Art6 by calcineurin activates endocytosis of the dicarboxylic amino acid permease Dip5 (but not that of the Gap1 permease), suggesting that the action of calcineurin on a given α-arrestin can affect the trafficking of specific cargo proteins [295]. More recently, it has been shown that calcineurin negatively regulates Aly1-mediated trafficking to the plasma membrane of the heterogously expressed mammalian potassium channel Kir2.1 [296].

It has been described that, during amino acid starvation, TORC2 promotes autophagy via its downstream target the protein kinase Ypk1, which inhibits calcineurin. In turn, calcineurin inhibits the activation of Gcn2, the eIF2α kinase and, consequently, the translational de-repression of the transcription factor Gcn4 [297]. These events are required for both initiation of the general amino acid control (GAAC) response and autophagy during amino acid starvation. More recently it has been proposed that Mid1, independently of Cch1, is required to maintain calcineurin active and prevent autophagy [298].

Early work showed that overexpression of calcineurin causes severe morphologic changes [299]. It was also found that treatment of cells with moderate doses of amiodarone, a drug that elicits an immediate influx of Ca^2+^, temporarily delayed cell cycle progression at different cell phases, being the Swe1-mediated delay in G_2_/M phase the one most dependent on calcineurin [300]. These and other evidences suggested a role for calcineurin in cell cycle regulation, usually in response to a variety of stresses, and in particular at the level of the morphogenesis checkpoint. Possibly, the characterization of the role of calcineurin in cell cycle and morphogenesis has been hampered by functional redundancy with other phosphatases, such as PP2A, as previously suggested [271]. However, recent work has shown that calcineurin is important in the regulation of the forkhead transcription factor Hcm1 [301]. This factor is involved in the expression of genes that are transcribed during S-phase and, in this way, it regulates chromosome segregation and is vital for maintaining genome stability. Hcm1 is activated by multiple phosphorylation at its C-terminal trans-activation domain by the cyclin-dependent kinase Cdk1, which also phosphorylates N-terminal residues that promote destabilization [302]. Upon stress, calcineurin would dephoshorylate the activating residues, while the destabilizing of phosphorylated sites would remain. In this way, the function of Hcm1 is abrogated, leading to a protective halt in cell cycle progression [303]. Interestingly, parallel evidences point to Slt2-mediated phosphorylation of the destabilizing N-terminal sites to decrease Hcm1 amounts in response to cell wall stress to delay proliferation [304].

**Figure 12:**
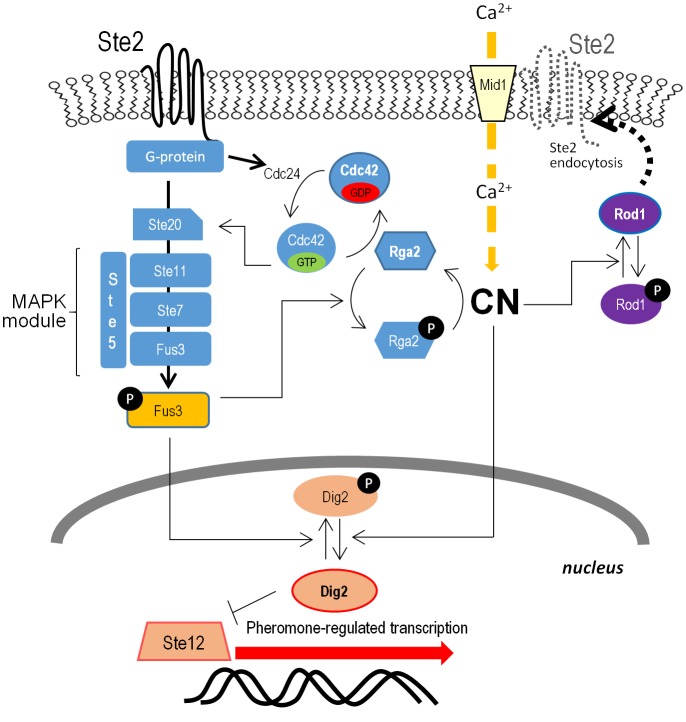
FIGURE 12: Roles of calcineurin during pheromone response. Upon stimulation with pheromone (α-factor in the model) entry of calcium promotes activation of calcineurin. To attenuate pheromone signaling, calcineurin acts at least at three levels: a) dephosphorylates Rod1 to promote endocytosis of the pheromone receptor; b) dephosphorylates Rga2, leading to decrease in Cdc42-GTP levels and attenuation of signaling through the Fus3 MAP kinase module; c) dephosphorylation and activation of the Ste12 inhibitor Dig2, to decrease pheromone-regulated specific gene transcription. Other targets downstream Fus3 are not included for simplicity.

Calcineurin plays an important role in down-modulating the response to mating pheromones as exemplified by the early finding that calcineurin mutants die upon prolonged exposure to the pheromone (**Figure 12**). During mating, the pheromone-activated receptor recruits the small GTPase Cdc24 to the membrane that, in its activated GTP-bound form, initiates a kinase cascade that leads to the activation of two partially overlapping MAPK, namely Fus3 and Kss1. Activation of these kinases (mainly Fus3) leads to phosphorylation of the repressor proteins Dig1 and Dig2, thus allowing the function of the Ste12 transcription factor and the consequent specific changes in gene expression pattern, which are accompanied by morphological changes (shmoo formation) and cycle arrest (see [305] for a review). Indeed, exposure to mating factor stimulates the Mid1/Cch1 high-affinity Ca^2+^ channel, inducing an increase in cytosolic Ca^2+^ that leads to calcineurin activation (**Figure 12**). The regulatory effect of calcineurin on the mating response occurs at different levels. From one side, calcineurin reduces the levels of pheromone receptor at the plasma membrane by dephosphorylating the arrestin Rod1/Art4 thus promoting endocytosis [306] while, in parallel, dephosphorylation of the Ste12 repressor Dig2 inhibits the pheromone-induced transcriptional response [263]. More recently, a third level of regulation has been proposed involving Rga2 [307], one of the Cdc42 GAPs (GTPase-activating protein). Rga2 is phosphorylated by Fus3 on inhibitory Ser and Thr sites, thus avoiding deactivation of Cdc42 upon pheromone signaling. Calcineurin would dephosphorylate Rag2, and reactivated Rag2 would promote transition of Cdc42 to its (inactive) GDP-bound form. In this way, calcineurin action would counteract Fus3-mediated downstream signaling in response to pheromone stimulus (**Figure 12**).

#### Calcineurin as virulence determinant in pathogenic fungi

The study of the calcineurin signaling pathway has received considerable attention in the last years since this enzyme seems to be a relevant virulence factor in major pathogenic fungi, such as *C. albicans, A. fumigatus* or *C. neoformans*. Thus, calcineurin has been proposed as an important drug target for antifungal treatments [308, 309]. The importance of calcineurin in virulence processes is quite widespread and has been also identified in fungal plant pathogens. Despite their very diverse morphological and physiological characteristics, in most cases the fungal processes affected by lack of calcineurin are the same: growth rate, morphological changes (often required for virulence, such as filamentation), and the ability to respond to stress conditions. It is worth noting that calcineurin has been found to be involved in cell wall maintenance in most fungal pathogens, and that this same process (specifically cell wall β-glucan synthesis) is the target of the lipopeptide drugs echinocandins. In contrast, calcineurin is necessary for survival at 37°C in some pathogens, as in *C. neoformans*, but not in other, such as *C. albicans* (see [310] and references therein).

Homologs of the Crz1 transcription factor can be found in pathogenic fungi (CrzA in *A. fumigatus*). Mutation of *CRZ1* does not fully reproduce the phenotypes caused by elimination of calcineurin, suggesting that other downstream targets relevant for virulence exist. Thus, calcineurin is necessary for growth in the presence of serum in *C*. *glabrata* and *C*. *albicans*, but Crz1 is not [311, 312]. The identification of a Crz1 homolog in *C. neoformans* has been somewhat controversial, but a recent work provided strong evidence that loss of Crz1 results in intermediate phenotypes compared to wild type and calcineurin mutant strains. This same work identified a subset of calcineurin-regulated genes that, in response to a 24 to 37°C shift, were not dependent on the presence of Crz1, as well as a smaller number of genes dependent on Crz1 but not on calcineurin [313]. A recent study demonstrated that in *Cryptococcus deneoformans* the initiation of the yeast-hyphal morphological transition is independent of Crz1, whereas the sporulation process is dependent on the transcription factor [314].

### PPT1 (PP5)

In *S. cerevisiae,* the Ppt1 phosphatase is 513-residue long protein with a distinct C-terminal half displaying the typical features of the PPP family and an N-terminal extension (~200 residues) that includes several TPR (tetratricopeptides repeats; four according the original paper, three upon our own analysis). The TPR repeats are structural motifs consisting of a degenerate 34 amino acid sequence often occurring in clusters and involved in protein-protein interactions. Ppt1 is well conserved across evolution and is found not only fungi, but also in plants and animals, including humans, named as PP5 [315].

A number of roles for PP5 in mammals were identified relatively soon (see [316] and references therein). In contrast, our knowledge on yeast Ppt1 was scarce for quite a few years. Ppt1 was present in both the nucleus and cytoplasm and was found to be expressed during logarithmic growth [317]. The same authors showed that although, as in PP5, Ppt1 activity was stimulated by lipids, the mammalian and yeast enzymes differed in several characteristics, such as the effect caused by removal of the TPR domain on Ppt1 activity. The lack of inhibitory effect upon removal of the TPR moiety was also observed during the characterization of the Ppt1 homolog from *Aspergillus oryzae* [318].

A major advance in understanding Ppt1 function was the finding that Hsp90 was specifically dephosphorylated by purified Ppt1 [319]. Hsp90 is a conserved ATP-dependent molecular chaperone involved in the conformational maturation of client proteins implicated in various and essential processes in eukaryotes. It was also shown that Ppt1 binds to Hsp90 through the phosphatase TPR domain and that lack of Ppt1 leads to hyperphosphorylation of Hsp90 *in vivo* and to a client-specific apparent decrease in the efficiency of the Hsp90 chaperone system [319]. Remarkably, Ppt1 also dephosphorylates the Hsp90 co-chaperone Cdc37 at Ser13 specifically when Cdc37 is associated with Hsp90 [320], and this dephosphorylation affects the activation of the Hsp90-Cdc37 protein kinase clients. Subsequent work by mass spectrometry revealed that Hsp90 is phosphorylated at multiple sites and that Ppt1 directly dephosphorylates two of these sites, S485 and S604 [321]. It is worth noting that interaction of PP5 and Hsp90 has been also observed in the plant *Arabidopsis thaliana* [322].

Investigation on modern sake yeast strains, which are highly sensitive to environmental stresses, revealed that the transcription factor Hsf1, relevant in the activation of multiple genes in response to various stresses, was constitutively hyperphosphorylated even in the absence of stress. Hsf1 hyperphosphorylation was caused by a spontaneous deletion of the *PPT1* locus [323]. Indeed, deletion of *PPT1* in a laboratory strain led to constitutive hyperphosphorylation of Hsf1, and introduction of functional *PPT1* derived from laboratory yeast recovered the HSE-mediated stress response of sake yeast. These findings suggested that Ppt1 is a positive regulator of Hsf1 [323]. Subsequently, it was found that Ppt1 directly interacts with Hsf1 via its TPR domain and that the phosphatase counteracts the CK2-dependent phosphorylation of the transcription factor on S608, which is an ethanol stress-specific repression mechanism of Hsf1 [324]. It must be noted that a functional relationship between Hsf1 and Hsp90 has been proposed, although there is some controversy about its nature [325].

Quantitative phosphoproteomic analysis using SILAC allowed specific comparison of wild type yeast cells and those carrying the *PPT1* deletion [326]. These authors found 33 phosphorylation sites significantly up-regulated in the mutant, corresponding to 28 different proteins (including Hsf1, Cdc37 and Hsp90), but no dephosphorylation events. In addition to proteins related to heat stress, the authors identified biosynthetic enzymes as particularly prominent among Ppt1-regulated phosphoproteins, suggesting still uncharacterized roles for Ppt1 in metabolic regulation.

### PP2C

**Figure 13:**
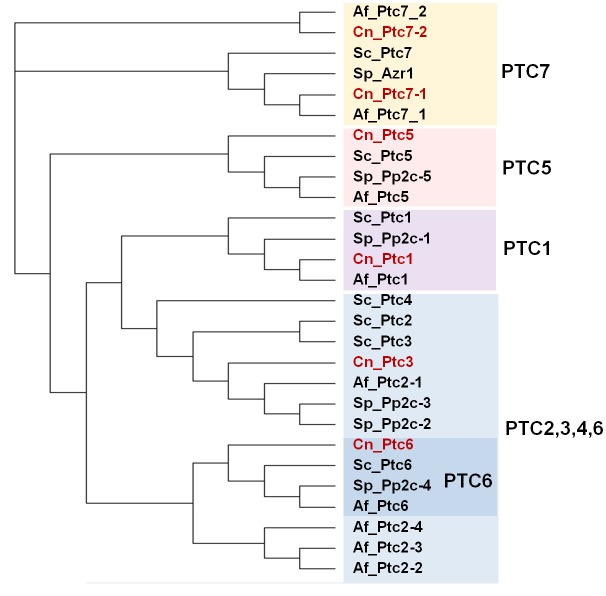
FIGURE 13: Phylogenetic relationships among PP2C phosphatases from various fungal species. Protein sequences correspond to organisms described in Figure 2. The analysis was performed as described in Figure 1.

Type PP2C phosphatases are represented by the family of Ptc proteins, included in the PPM group of phosphatases since their activity depends on metal ions. In *S. cerevisiae*, this family is encoded by 7 different genes (**Figure 13**), ranging from *PTC1* to *PTC7*. Not all members are represented in all fungi and, in some species, more than one gene may exist for a specific Ptc [327]. In contrast to other PPP's the catalytic subunits of the PP2C family are not usually associated with regulatory subunits. Evidence has been reported that deletion of all seven Ptc-encoding genes is not lethal [328].

### Ptc1 phosphatase

Fungi Ptc1 constitute a well-defined group of proteins that are involved in many cellular functions often not shared by other members of the PPM family [327, 329]. In *S. cerevisiae*, cells lacking Ptc1 show a large number of functional defects, including sensitivity to high pH, LiCl, CaCl_2_, ZnCl_2_, CFW, caffeine, rapamycin, and inability to grow on ethanol as the only carbon source. These mutants also show defects in budding pattern, vacuolar morphology or inheritance of cellular organelles (see [327] and references therein). Ptc1 also plays a role in the TOR pathway and in cation homeostasis, among other functions.

#### Ptc1 and MAP kinase pathways

A role of Ptc1 in regulating MAP kinase pathways was established long time ago. Thus, the regulation of the high-osmolarity glycerol (HOG) pathway is achieved by dephosphorylation of Hog1 MAP kinase, and this is accomplished by binding of the phosphatase to the N-terminal domain of the adaptor protein Nbp2, which in turn interacts through a SH3 domain with the scaffold protein Pbs2 [330].

A number of independent evidences placed Ptc1 in close relationship with the CWI pathway, including the observation that mutation of *PTC1* results in increased expression of genes induced by cell wall damage in a way that was dependent on the Slt2 MAPK module [329]. Therefore, lack of Ptc1 mimicked the activation of the CWI pathway. Recently, it has been shown that Ptc1 specifically dephosphorylates and inactivates Mkk1, one of the two MAP kinase kinases upstream Slt2 [331]. Remarkably, many phenotypic defects described for the *ptc1* mutant can be attributed to the abnormal activation of the Slt2 pathway, as a result of the incapacity to properly dephosphorylate and down-regulate Mkk1, indicating that the regulation of the CWI pathway is a major cellular role for Ptc1 [331]. There is some evidence that Ptc6 might contribute, although to a much lesser extent, to the regulation of the CWI pathway (see below). In addition to mediate the interaction between Ptc1 and the HOG pathway, Nbp2 also functions as a negative regulator of the CWI pathway by facilitating the interaction of Ptc1 with Bck1. This role of Nbp2 in Ptc1 signaling might be extended to other MAPK pathways and seems to be conserved across the Ascomycete species.

Ptc1 is also involved in the MAPK pathway activated by pheromone, regulating the mechanism that controls the switch-like mating decision [332]. Ptc1 and the MAPK Fus3 compete for the control of the phosphorylation state of Ste5, the scaffold protein of this MAP kinase module. Dephosphorylation of Ste5 is a requisite for full relief and dissociation of the Fus3-Ste5 complex, which leads to the mating response. Lack of Ptc1, thus, prevents shmooing and reduces activation of Fus3.

#### Ptc1 and cation homeostasis

Diverse evidences indicate that, among Ptc enzymes, Ptc1 is the major responsible for Li^+^ tolerance, as only *ptc1* single mutants display increased sensitivity to this cation. It has been determined that Ptc1 is necessary for the proper extrusion of Li^+^ since *ptc1* mutant cells accumulate higher concentrations of this cation due to a decreased expression of the Na^+^-ATPase *ENA1* gene. Evidences suggest a possible role of Ptc1 in the regulation of Ppz1/2-Hal3 (see PPZ section above) that could be exerted through the Hal5 protein kinase [333, 334].

A systematic analysis performed with all possible combination of *ptc* disruptions showed that only Ptc1 and Ptc2 and/or Ptc4 had a redundant role in the tolerance at high concentrations of Na^+^ ions in the medium [328]. A strain containing *ptc2 ptc3 ptc5 ptc7* simultaneous deletions tolerates 1.5 M NaCl similarly to wild type cells, but the same strain was unable to grow in the presence of 0.4 M LiCl (and grew poorly in YPD medium supplemented with adenine). The same report showed that the Li^+^-sensitive phenotype displayed by the *ptc1* mutant cells is suppressed by additional deletion of *PTC7* [328], although no explanation was proposed for this effect.

#### Ptc1 as regulator of the cell cycle at the G2/M transition

Several recent evidences support a functional positive role of Ptc1 in the G2/M transition. Under stress conditions activating the CWI pathway *ptc1* mutant cells display hyperphosphorylated Cdc28 kinase and reduced Clb2-associated Cdc28 activity, and they accumulate with duplicated DNA, pointing out to a delay in the G2/M transition [331, 335]. Consistently, overexpression of *MIH1*, coding for the Swe1 tyrosine phosphatase that promotes progression through cell cycle, suppressed sensitivity of *ptc1* mutant cells to cell wall stressors, whereas deletion of *MIH1* increased the sensitivity of *ptc1* mutant cells to CFW [335]. Also, overexpression of *PPH22* or *ZDS1*, important actors in Mih1 regulation, are able to suppress certain defects of the *ptc1* mutant [335]. These cell cycle-related alterations also are attenuated by mutation of the *MKK1* gene, encoding a MAP kinase kinase upstream Slt2, leading to the proposal that their primary origin is the hyperactivation of the Slt2 pathway described above.

#### Other functions of Ptc1

Several evidences pointed out that *ptc1* mutant cells were hypersensitive to rapamycin, indicating a possible functional connection between Ptc1 and the TOR pathway (see [327] and references therein). In fact, lack of Ptc1 impairs TOR-mediated signaling on Gln3 and Msn2/4 transcription factors, leading to a general attenuation of the transcriptional changes triggered by rapamycin. These defects are, in part, caused by the low levels and impaired dephosphorylation of Tip41 observed in *ptc1* mutant cells. Hyperphosphorylated Tip41 cannot bind to nor inhibit Tap42, which is required for normal signaling through the pathway involving inhibition of Sit4 [218]. It has been shown [185] that, in contrast to Gln3, rapamycin-elicited Ure2 dephosphorylation occurred independently of Ptc1 (as well as of Sit4 and Pph21/22, Siw14 and Ppz1).

Ptc1 has been described as one of the phosphatases, together with Glc7-Reg1 and Sit4, directly or indirectly participating in the glucose-dependent dephosphorylation of the activation loop (Thr210) of Snf1, the yeast AMPK [26] when growing on medium containing high glucose concentrations. This seems to be a conserved function of the PP2C, since the close human orthologs Ppm1E, and probably PpmF, are implicated in the dephosphoryation of human AMPK [336].

The inheritance of several cellular organelles is a process in which Ptc1, together with the adaptor protein Nbp2, is involved and it has been extensively reviewed [327]. More recently, Ptc1 was found to promote the association of myosin-V, encoded by *MYO2* and *MYO4*, with its organelle-specific adaptor proteins such as Mmr1, Vac17 and Inp2, being important for the proper inheritance of mitochondria, vacuole and peroxisomes, respectively [337, 338]. The phosphorylated form of Mmr1 has been found increased in cells lacking Ptc1 [338]. In the same cells, the steady-state level of Mmr1, Vac17 and Inp2 was reduced [337].

### The Ptc2, Ptc3, and Ptc4 phosphatases

The analysis of the primary structure of these proteins denotes that they are closely related, sharing one only branch where two main groups of proteins can be differentiated: Ptc2/3 and the Ptc4 (**Figures 1** and **13**). The number of genes coding for proteins in any of these groups varies in different fungi species, ranging from none (in the case of *Tuber melanosporum*) to three. *S. pombe, Schizosaccharomyces japonicus, C. glabrata* and *S. cerevisiae* are the only species analyzed containing two genes encoding the closely related Ptc2/Ptc3. On the other side, Ptc4-related proteins are specific of fungi included in the family of *Saccharomycetaceae*, being absent in the rest of the *Saccharomycetales*.

Although Ptc2/Ptc3, as Ptc1, are involved in the regulation of the HOG MAP kinase pathway, they do not interact with Nbp2 and, since they cannot replace Ptc1 (see [327] and references therein), they are probably involved in different cellular roles. As in the case of Ptc1, Ptc2 and Ptc4 have recently been identified as phosphatases that physically interact to and regulate Slt2, the MAPK of the CWI pathway [50].

It has been suggested that Ptc2/3 act as negative regulator of the UPR by interacting to the ER membrane localized Ire1, a protein with Ser/Thr protein kinase and RNase activities that triggers the UPR. Ptc2/3 attenuate the signaling by decreasing the phosphorylation level of Ire1 and its activation which, in turn, decreases the levels of the *HAC1* cytosolic splicing [29, 339].

This subfamily of PP2C were identified as phosphatases required for the recovery from the DNA DSB since, in the absence of Ptc2 and Ptc3, Rad53 remains hyperphosphorylated even after a DSB is repaired. In the suggested model, Ptc2 is phosphorylated by CKII and thereby dephosphorylates Rad53 [189]. In *S. cerevisiae*, cells deleted in *PTC2, PTC3* and *PPH3* display defects in repairing a HO endonuclease-induced DSB. This phenotype is not observed in *ptc2 ptc3* double mutant cells [182], suggesting that Ptc2/3 are dispensable for both, Rad53 deactivation and checkpoint recovery after replication stress and for the dephosphorylation of Rad53 that has been activated in S phase in response to DNA methylation.

### The Ptc5, Ptc6, and Ptc7 phosphatases

By using classical genetics and biochemistry techniques Ptc5 and Ptc6 were identified as mitochondrial proteins acting on the pyruvate dehydrogenase complex (PDC), specifically on the E1 subunit Pda1 [see [327] for references]. The activity of the PDC is important for regulating the entry of pyruvate into the TCA cycle and, consequently, the overall maintenance of cellular glucose homeostasis. More recently, using comparative mitochondrial phosphoproteomics and analyses of protein–protein interactions by affinity enrichment–mass spectrometry, Ptc6 (also known as Aup1) has been identified as the primary PDC phosphatase in *S. cerevisiae* and the only able to dephosphorylate Pda1 *in vitro* [340]. According to this study, Ptc5 and Ptc7 have other distinct, non-overlapping functions, such as dephosphorylation of the glycerol-3-phosphate dehydrogenase Gpd1.

Ptc6 has been found necessary for survival of stationary phase cells and it has recently been found involved in mitophagy, the autophagic degradation of mitochondria, which is an important housekeeping process in eukaryotic cells [341]. It was subsequently established that in Ptc6-deficient cells the retrograde signaling pathway (RTG) was defective, and the phosphorylation pattern of Rtg3, the transcription factor mediating RTG response, was altered. This allowed proposing that the role of Ptc6 in mitophagy could be explained through regulation of Rtg3-dependent transcription [342].

Cells lacking Ptc6 (but not the *ptc5* mutant) are sensitive to rapamycin and caffeine [218], and this phenotype is independent of the alteration in the PDC activity [343]. In fact, the transcriptional profile of the *ptc6* mutants was more similar to that of the *ptc1* strain than to other Ptc-deficient cells, including *ptc5*, albeit the sensitivity to rapamycin is not rescued by overexpression of *PTC1*. It was also found that this mutant suffers a considerable attenuation in its transcriptional response to rapamycin, being particularly affected the subset of repressed genes encoding ribosomal proteins or involved in rRNA processing [343]. The sensitivity of *S. cerevisiae ptc6* mutants to rapamycin and caffeine is complemented by low-copy expression of the *C. albicans* Ca*PTC6* gene, although deletion of Ca*PTC6* does not alter *C. albicans* sensitivity to these compounds [344]. Lack of Ptc6 renders cells tolerant to cell wall damaging agents, such as Congo Red or CFW [343], although it has been hypothesized that this effect might be strain-specific [345].

Ptc7 (YHR076w) was biochemically identified as a protein phosphatase more than 15 years ago and a mitochondrial localization was initially reported [346]. It was subsequently found that the gene contains a functional intron, very near to the N-terminus, that lacks a consensus branch-point sequence [347]. This intron could be spliced out or retained, producing in the first case a slightly shorter version (Ptc7_s_) that is targeted to the mitochondria and, in the second case, a version (Ptc7_u_) that was localized to the nuclear envelope. Targeting to the nuclear envelope has been attributed to a predicted trans-membrane helix encoded by the retained intron. Both versions retain the PP2C catalytic domain, and the prevalence of a given isoform is determined by the carbon source, being Ptc7_u_ more prominent when cells were grown in fermentable media [347]. The transmembrane domain and the mitochondrial targeting signal are largely conserved between *Saccharomycetaceae PTC7* orthologs and are mutually exclusive. Interestingly, there is at least one example (the budding yeast *Tetrapisispora blattae)* of the existence of two individual genes, one encoding a predicted nuclear envelope-associated Ptc7, while the other encodes a predicted mitochondrial phosphatase [348].

It has been reported that the mitochondrial version of Ptc7 activates coenzyme Q (CoQ, also known as ubiquinone) biosynthesis by dephosphorylation of the demethoxy-Q6 hydroxylase Coq7, a key regulatory point in the yeast CoQ biosynthetic pathway [349]. In this way, Ptc7 promotes respiratory metabolism. The impact of the absence of Ptc7 in the phosphorylation status of mitochondrial proteins was recently analyzed [350]. Nearly 20 mitochondrial proteins increased their phosphorylation levels, including the citrate synthase Cit1. It was found that hyperphosphorylation of Cit1 at S362 disrupted the enzyme function and that this residue was dephosphorylated *in vitro* by Ptc7. The authors suggested that, by regulating citric acid production, Ptc7 might impact not only in the functioning of the TCA cycle, but also might influence *de novo* fatty acid synthesis. Remarkably, they found that lack of Ptc7 did not alter CoQ levels. It must be noted, however, that the methodologies and growth conditions used in both apparently contradictory studies largely differ. A very recent report [351] has shown that the lack of Snf2, the catalytic subunit of the SWI/SNF chromatin remodeling complex, alters the relative levels of Ptc7_s_ and Ptc7_u_ isoforms, promoting predominance of the spliced Ptc7_s_ isoform. The increase in *PTC7* splicing was attributed to down-regulation of the very abundant transcripts of the genes encoding ribosomal proteins, leading to an increase in the available pool of spliceosomes. The authors found that exclusive expression of Ptc7_s_ increased CoQ synthesis, whereas exclusive expression of Ptc7_u_ had the opposite effect. This would explain the observed increase in both the rate of synthesis and steady-state levels of CoQ in Snf2-deficient cells and might contribute to justify the contradictory results on CoQ production described above. Lack of Ptc7 has been also related to the shortening of the chronological life span (CLS) and prevention of mitophagy, whereas overexpression of the protein had the opposite effects [352]. Since CLS shortening could not be rescue by exogenous yeast CoQ supplementation, this was additional evidence pointing out that Ptc7 could function beyond the regulation of CoQ biosynthesis. It is worth noting that a very recent report [353] has shown that PPTC7, the human orthologue of yeast Ptc7, rescues CoQ deficiency in yeast and that phosphorylated recombinant human COQ7 is an *in vitro* substrate for PPTC7. In addition, modulation of PPTC7 levels did result in changes in CoQ content in human cells, suggesting that the regulatory role of Ptc7 enzymes could have a very broad distribution. Collectively, all these findings point to Ptc7 being an important regulatory phosphatase in mitochondrial metabolism.

### PP2c's in other fungi

Most of the current knowledge about PP2C proteins in other fungi has previously been reviewed [327], so we will focus here in the most recent findings.

Fungal Ptc1 proteins seem to be required for pathogenesis in both, animal and plant pathogenic fungi (see [327] for references). BcPtc1 or BcPtc3 proteins of the Ascomycota fungus *Botrytis cinerea*, a plant pathogen, are required for growth in the presence of osmotic and oxidative stressors, and cell wall degrading enzymes. Lack of these proteins also showed decreased virulence on different host plant tissues [354]. In addition to Ptc1, Ptc3 has been found to be involved in virulence in the cereal crops pathogen *Fusarium graminearum* [355].

In fission yeast, SpPtc4 plays a major role in inactivating the mitochondrial pool of the stress-activated MAP kinase Sty1, most similar to ScHog1, specifically upon oxidative but not under other types of stress. SpPtc4 is strongly associated with mitochondrial membranes and is targeted there by an N-terminal mitochondrial targeting sequence (MTS), which is cleaved upon import. Cleavage of the SpPtc4 MTS is greatly reduced specifically upon oxidative stress, resulting in the full-length form of the phosphatase that displays a stronger interaction with Sty1. Consistently, the mitochondrial pool of Sty1 is hyperactivated in the absence of SpPtc4 [356].

## FINAL REMARKS

One of the most important challenges in this field is to identify and characterize the physiological substrates of phosphatases. This objective presents several difficulties. As in the case of protein kinases, a given protein may be the substrate of a phosphatase only for a short period (i.e., when cells are in a definite physiological state or under a specific stress). An additional difficulty is that some phosphatases may have different substrates depending on which regulatory subunit they bind to. Similarly, some enzymes could likely exhibit a high degree of functional redundancy (such as Pph21/Pph22, Ppz1/Ppz2, or Ptc2/Ptc3/Ptc4). Unlike protein kinases, massive techniques for *in vitro* determination of phosphatase substrates require the substrates be previously phosphorylated and, as far as we know, the conditions and/or kinases required for the phosphorylation of all possible substrates are still unknown. For these reasons, it seems plausible that the next steps for the identification of (direct or indirect) substrates will be based on detecting the overall changes in the phosphorylation state of proteins when cells are under specific cell growth conditions, either in the absence of one (or several redundant) specific phosphatases, or overexpressing a given enzyme.

## SUPPLEMENTAL MATERIAL

Click here for supplemental data file.

All supplemental data for this article are available online at http://www.microbialcell.com/researcharticles/2019a-arino-microbial-cell/.

## Abbreviations:

AID: – autoinhibitory domain,
CDRE: – calcineurin-dependent response element,
CFW: – Calcofluor White,
CLS: – chronological lifespan,
CNA: – calcineurin A,
CoQ: – coenzyme Q,
CWI: – cell wall integrity,
DSB: – double strand break,
eIF: – eukaryotic translation initiation factor,
GAP: – GTPase-activating protein,
HOG: – high-osmolarity glycerol,
MEN: – Mitotic Exit Network,
MTS: – mitochondrial targeting sequence,
NCR: – nitrogen catabolite repression,
PDC: – pyruvate dehydrogenase complex,
PPases: – protein phosphatases,
PPCDC: – phosphopantothenoyl-cysteine decarboxylase,
PTPA: – Phosphotyrosyl Phosphatase Activator,
RCAN: – regulator of calcineurin,
SAC: – Spindle Assembly Checkpoint,
SAM: – S-adenosylmethionine,
SAP: – Sit4-associated protein,
SIN: – Septation Initiation Network,
STRIPAK: – STRiatin-Interacting Phosphatases And Kinases,
TCA: – tricarboxylic acid,
TORC: – Target of Rapamycin Complex,
TPR: – tetratricopeptides repeats,
UPR: – unfolded protein response.
