# Supplementary material for: Ser/Thr protein phosphatases in fungi: structure, regulation and function
Source: Microb Cell. 2019 Apr 24;6(5):217–56. doi: 10.15698/mic2019.05.677 (PMC6506691; doi:10.15698/mic2019.05.677)
Supplement: Supplementary file 1 [file mic-06-217-s01.pdf]

Ariño et al.

Supplemental Table 1

Protein sequences used for the construction of phylogenetic trees

**PP1**	
Sp_PpZ	sp|P78968.1|
Cn_Ppz1	XP_012047378.1
Sc_Ppz2	NP_010724
Sc_Ppz1	NP_013696.1
Af_Ppz	XP_749527.1
Sc_Ppq1	NP_015146.1
Sp_Pp1-2	sp|P23880.1|
Sc_Glc7	NP_011059.3
Af_Pp1	XP_750244.1
Sp_Pp1-1	sp|P13681.1|
Cn_Pp1	XP_012047402.1
**PP2A**	
Sp_Ppg	sp|Q10298.1|
Sc_Ppg1	NP_014429.3
Cn_Ppg1	XP_012049795.1
Af_Ppg1	XP_753450.2
Sc_Pph22	NP_010093.1
Sc_Pph21	NP_010147.1
Sp_Pp2a-2	sp|P23636.1
Sp_Pp2a-1	sp|P23635.1|
Cn_Pph22	XP_012049994.1
Af_Pph21	XP_750971.1
Cn_Sit4	XP_012048933.1
Sp_Ppe1	sp|P36614.1|
Sc_Sit4	NP_010236.1
Af_SitA	XP_751030.2
Cn_Pph3	XP_012046699.1
Sp_Pph3	sp|O74789.1|
Af_Pp2a	XP_753450.2
Sc_Pph3	NP_010360.1
**PP2B**	
Sc_Ppt1	NP_011639.3
Sp_Ppt1	sp|O43049.2|
Af_Ppt1	XP_753963.1
Cn_Ppt1	XP_012052342.1
Sc_Cmp2	NP_013655.1
Sc_Cna1	NP_013537.1
Sp_Pp2B	sp|Q12705.2|
Af_Pp2B	XP_753703.1
Cn_Cmp2	XP_012052213.1

**PP2C**	
Af_Ptc7_2	XP_750014.1
Cn_Ptc7-2	XP_012048589.1
Sc_Ptc7	NP_011943
Sp_Azr1	sp|Q09189.3
Cn_Ptc7-1	XP_012051325.1
Af_Ptc7_1	XP_747265.1
Cn_Ptc5	XP_012046308.1
Sc_Ptc5	NP_014733.1
Sp_Pp2c-5	p|O14189.4
Af_Ptc5	XP_750435.1
Sc_Ptc1	NP_010278.3
Sp_Pp2c-1	sp|P40371.1|
Cn_Ptc1	XP_012046759.1
Af_Ptc1	XP_753320.1
Sc_Ptc4	NP_009683
Sc_Ptc2	NP_011013.1
Sc_Ptc3	NP_009497.2
Cn_Ptc3	XP_012047784.1
Af_Ptc2-1	XP_752295.1
Sp_Pp2c-3	sp|Q09173.1|
Sp_Pp2c-2	sp|Q09172.1
Cn_Ptc6	XP_012053325.1
Sc_Ptc6	NP_010003
Sp_Pp2c-4	sp|O14156.2
Af_Ptc6	XP_752946.1
Af_Ptc2-4	XP_746384.1
Af_Ptc2-3	XP_753280.1
Af_Ptc2-2	XP_749521.1
